# Topological data analysis of thoracic radiographic images shows improved radiomics-based lung tumor histology prediction

**DOI:** 10.1016/j.patter.2022.100657

**Published:** 2022-12-12

**Authors:** Robin Vandaele, Pritam Mukherjee, Heather Marie Selby, Rajesh Pravin Shah, Olivier Gevaert

**Affiliations:** 1 Department of Applied Mathematics, Computer Science and Statistics, Ghent University, 9000 Ghent, Belgium; 2 Data Mining and Modeling for Biomedicine, VIB Inflammation Research Center, 9052 Ghent, Belgium; 3 IDLab, Department of Electronics and Information Systems, Ghent University, Gent, Belgium; 4 Stanford Center for Biomedical Informatics Research (BMIR), Department of Medicine, Stanford University School of Medicine, Stanford, CA 94305, USA; 5 Department of Biomedical Data Science, Stanford University School of Medicine, Stanford, CA 94305, USA; 6 Veterans Affairs Palo Alto Health Care System, Palo Alto, CA, USA; 7 Department of Radiology, Stanford University, Stanford, CA, USA

**Keywords:** topological data analysis, radiology, histology, computed tomography, computer vision, supervised learning, feature engineering

## Abstract

Topological data analysis provides tools to capture wide-scale structural shape information in data. Its main method, persistent homology, has found successful applications to various machine-learning problems. Despite its recent gain in popularity, much of its potential for medical image analysis remains undiscovered. We explore the prominent learning problems on thoracic radiographic images of lung tumors for which persistent homology improves radiomic-based learning. It turns out that our topological features well capture complementary information important for benign versus malignant and adenocarcinoma versus squamous cell carcinoma tumor prediction while contributing less consistently to small cell versus non-small cell—an interesting result in its own right. Furthermore, while radiomic features are better for predicting malignancy scores assigned by expert radiologists through visual inspection, we find that topological features are better for predicting more accurate histology assessed through long-term radiology review, biopsy, surgical resection, progression, or response.

## Highlights

• We study all of the primary lung tumor classification problems
• We illustrate persistent homology for computed tomography scan images
• We show that topological features may improve radiomic-based histology prediction
• We compare predicting from radiologists’ and more accurate histology assessment

## The bigger picture

We study the use of automated features from topological data analysis for all of the most prominent lung tumor histology prediction problems from thoracic images. This is significant because machine learning from computed tomography images requires effective feature engineering methods due to their vast three-dimensional structure and the common small sample sizes in medical cohorts. This is important for rapid diagnosis, intervention, customized treatment, and monitoring of patients. The state of the art considers radiomic features. However, we find that extending these with topological information may improve the accuracy of diagnosis. Finally, while radiomic features appear to be more appropriate for mimicking a radiologist’s visual assessment, it turns out that topological features allow more accurate histology prediction, as confirmed through review of radiology images to show 2 years of stable nodule, biopsy, surgical resection, progression, or response.

## Teaser

We find that topology, a mathematical subject that considers the study of shape, allows one to quantify important information in CT scan images of lung tumors. In this way, tumoral patterns and characteristics that fail to be discovered by the naked eye are discovered. From these, one is able to more rapidly and accurately diagnose the tumor type, which is important for intervention, customized treatment, and monitoring of patients.

## Introduction

The recent rise of quantitative imaging in medicine led to new opportunities for assessing severity, change, and disease through quantifiable features from medical images.^1^^,^^2^ In particular, determining lung cancer histology from computed tomography (CT) scan images is a crucial problem in medical image analysis. Machine-learning models can lead to rapid diagnosis, intervention, customized treatment, and monitoring of patients with lung cancer from such images, reducing the effects of human error in the clinical decision-making process. State-of-the-art models are often based on radiomic features, which cover a wide range of quantitative tumor characteristics such as lesion shape, location, and vascularity.^3^

Complementary to this, the rising field of topological data analysis (TDA)^4^ and, in particular, its main method, persistent homology,^5^ provide an unparalleled tool to quantify local to global structural information in data. Persistent homology constructs a time-parameterized sequence of combinatorial structures—which can be seen as higher-order generalizations of graphs—from the input data and tracks changes in the topological features—more precisely, holes such as connected components, loops, and voids—along this sequence. These changes are summarized by persistence diagrams, which are sets D of two-dimensional point coordinates (b,d), marking that a topological feature occurred from birth time b until death time d in the sequence. Persistence diagrams have been effectively incorporated into learning from topological information for various biomedical machine-learning problems. This includes tasks such as predicting protein-protein interaction binding affinity changes,^6^ biomedical network classification,^7^^,^^8^ survival prediction of patients with cancer,^9^^,^^10^ and skin lesion segmentation.^11^ We emphasize that beyond the short introduction to persistent homology given in this paragraph, the many technical and mathematical concepts on which TDA is founded and which are inherent to its introduction are omitted from the main paper. However, a high-level and comprehensive introduction to persistent homology and persistence diagrams that, in particular, focuses on medical image analysis of lung tumors, and which explains how we may consecutively engineer the topological features that are used in this paper, is provided in the supplemental information.

In this article, we study the use of TDA for lung tumor histology prediction from thoracic radiographic images. Our main goal is to study the added value of TDA to all of the prominent learning problems on lung tumor CT scan images compared with state-of-the-art quantitative imaging tools.^12^^,^^13^^,^^14^^,^^15^^,^^16^

**Figure 1:**
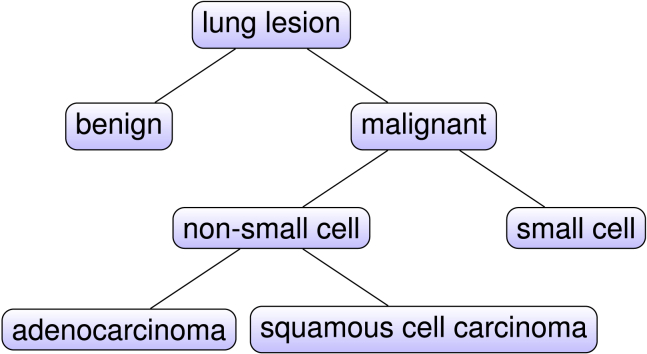
The main hierarchical structure of lung lesions Each pair of siblings with the same parent in the tree induces a binary classification problem that we study in this article.

**Figure 2:**
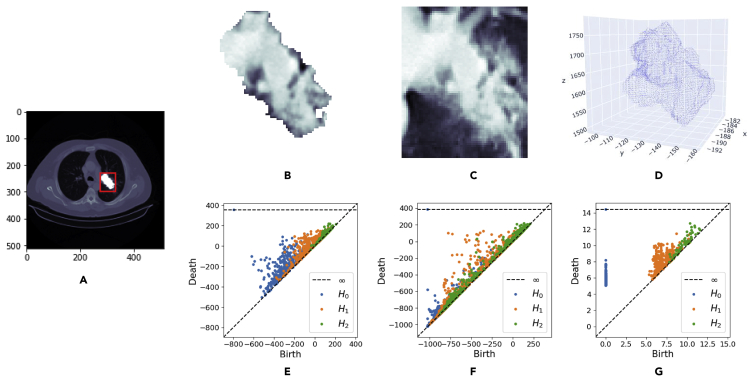
Different ways to compute persistent homology from thoracic radiographic images (A) An example 2D slice of a lung tumor CT scan. Pixel values—which can range on a grayscale from black to white—capture the radiodensity of the material at the location. Brighter pixels correspond to radiodense material that more inhibits the passage of radiation (X-rays), such as bones or tumor tissue. Darker pixels correspond to radiopaque material that allows radiation to pass more freely, such as muscle and skin. The tumor in the lungs is marked by a red boundary box. The segmented tumor pixels are highlighted in white, not to be confused with their CT pixel values or, thus, radiodensity values, which are better visualized in the following two images. (B) The same slice of the CT scan image but restricted to the segmented tumor pixels. (C) The same slice of the CT scan image but restricted to the pixels in the boundary box of the segmented tumor. (D) A point cloud representing the tumor surface in $R^{3}$. It can be defined by stacking the tumor contours of all 2D slices of the CT scan. (E) The persistence diagrams obtained from the sublevel filtration of the 3D tumor image, of which a 2D slice is shown on image (B) above. Three persistence diagrams are plotted on top of each other. There is one diagram for each of the three dimensions of topological hole considered (H0/0-dim.: components, H1/1-dim.: cycles, H2/2-dim.: voids). (F) The persistence diagrams obtained from the sublevel filtration of the 3D tumor image with surrounding boundary box pixels, of which a 2D slice is shown in (C) above. (G) The approximated persistence diagrams for the Vietoris-Rips filtration of the point cloud representing the tumor surface in $R^{3}$, shown in (D) above.

This retrospective study was approved by the Institutional Review Board overseeing research at both the VA Palo Alto Health Care System and Stanford University. All CT images were obtained from both the Palo Alto and San Francisco VA Picture Archiving and Communication Systems (PACS). We obtained chest CT studies exhibiting cancerous and benign nodules between December 2015 and 2018. For the cancer studies, the inclusion criteria were presence of small cell lung cancer (SCLC), adenocarcinoma (ADC), or squamous cell cancer (SCC). A discussion of the inclusion, exclusion, and size criteria has been previously described.^16^ The same criteria were utilized for the San Francisco VA cohort. A CT scan of a primary lung tumor was obtained from each patient, and diagnoses were obtained through biopsy, resection, or serial follow up. Table 1 gives an overview of the number of patients per tumor type. Furthermore, the tumor in each scan was manually delineated by an expert radiologist with greater than 10 years of experience using ITK-SNAP.^17^ Figure 2A shows an example of the annotation. Figure 1 displays our three classification problems of interest: each pair of siblings with the same parent in the tree induces a binary classification problem.

**Table 1:** The number of observations for each class of lung tumor in the data, with and without added contrast, in the San Francisco/Palo Alto (SF/PA) cohort and the Lung Image Database Consortium (LIDC)

	With contrast	Without contrast	Total
SF/PA			
benign	22	62	84
malignant	33	47	80
small	17	10	27
non-small	16	37	53
adeno	11	20	31
squamous	5	15	20
total	55	109	164
LIDC			
benign	24	5	29
malignant	17	8	25
total	41	13	54

Note that the classes in the SF/PA cohort are not mutually distinct (Figure 1). Here, the last row thus does not equal the sum of the column values.

Next, we used CT images from 2,544 lung tumor nodules in the Lung Image Database Consortium (LIDC) image collection.^18^^,^^19^^,^^20^ These nodules include both primary lung tumors as well as metastatic cancers originating from non-lung-tumor sites. The nodules are divided into 807 that were obtained from a scan with contrast material and 1,737 without. The data also contain nodule segmentations provided by multiple expert radiologists for each scan. For tumors with multiple annotations, we used the 50%-consensus segmentation, from which we obtained radiomic and topological features, which will be introduced below. Each annotation included a malignancy score on a discrete scale from 1 to 5 assigned by the expert radiologists. The mean of malignancy scores from the different annotations was considered the “consensus score” for each nodule. Besides the malignancy scores, the radiologists also assigned scores for eight semantic features (SEM): subtlety, internal structure, calcification, sphericity, margin, lobulation, spiculation, and texture.

Finally, we considered the histological diagnosis for benign versus malignant tumor classification on a smaller LIDC data sample of 54 primary lung tumor nodules for which true diagnoses were available. These were obtained based on one of the following criteria: review of radiology images to show 2 years of stable nodule, biopsy, surgical resection, progression, or response.^18^^,^^19^^,^^20^ Table 1 summarizes the number of patients per primary tumor type, with and without added contrast material, for which diagnoses were available.

The main contributions of this work are as follows. Through a cohort of patients with lung cancer from multiple institutes including Stanford and several VA hospitals, we compare topological-features-based classifications with standard radiomic-features-based classifications of lung tumors with and without contrast material. In particular, we study all of the main lung tumor classification problems that can be considered: “benign versus malignant,” “small cell versus non-small cell,” and “ADC versus squamous cell carcinoma” (Figure 1). We show that topological features consistently provide additional and valuable information when combined with radiomic features for benign versus malignant classification and adeno versus squamous while, interestingly, contributing less consistently to small cell versus non-small cell. Furthermore, the enhanced performance for malignancy prediction is confirmed for both a binary and a continuous outcome on the LIDC image collection.^18^^,^^19^^,^^20^ Finally, we discuss further directions for studying and improving lung tumor histology prediction through TDA.

## Results

All results for the various histology prediction problems are summarized in Table 2. Rows correspond to the considered classification or regression problem. More detailed tables can be found in the supplemental information (Tables S1–S10). We also include correlation matrices as well as feature importances for the features selected by the pipelines including a logistic/linear regression (LR) model (Figures S3–S12), which were consistently among the better-performing models, either on their own or through a soft-voting ensemble.

**Table 2:** Mean performances in percentage (ROC AUC for classification and $r^{2}$ for regression) for lung tumor histology prediction

Problem	C	SEM	Rad	Top	Concat	Vote	Stack	Best model	Best score	p vote ≥ rad
Benign versus	Y	–	84.6	86.8	86.7	87.9^a^	85.8	LR + vote	88.9	$5.7\cdot 10^{-5}$
malignant (SF/PA)	N	–	74.0	75.7	76.5	78.2^a^	73.8	LR + vote	80.2	$1.7\cdot 10^{-7}$
Small cell versus	Y	–	77.5^a^	62.7	66.1	75.0	71.7	LR + rad only	79.8	0.94
non-small cell (SF/PA)	N	–	80.6	78.6	80.9	83.4^a^	75.9	RF + vote	86.8	$3.9\cdot 10^{-2}$
Adeno versus	Y	–	67.2	91.2^a^	90.1	88.3	–	RF + top/concat	98.3	$1.2\cdot 10^{-17}$
squamous (SF/PA)	N	–	64.3	70.0	68.8	71.2^a^	65.1	BAY + vote	75.0	$3.8\cdot 10^{-5}$
Malignancy	Y	61.1	56.3	52.0	53.4	59.0^a^	53.5	RF + vote	61.3	$5.6\cdot 10^{-7}$
regression (LIDC)	N	54.2	42.8	36.4	38.2	45.8^a^	38.8	RF + vote	49.0	$3.3\cdot 10^{-9}$
Benign versus	Y	66.9	58.2	61.6^a^	59.3	60.1	56.6	KNN + stack	67.7	0.11
malignant (LIDC)	N	15.6	54.1	63.1	66.2^a^	61.5	43.3	XGB + vote	78.0	$1.6\cdot 10^{-2}$

C, whether contrast material was added (Y) or not (N); SEM, semantic features that were manually assigned by expert radiologists; rad, radiomic features; top, topological features; concat, concatenated radiomic and topological features; vote, voting ensemble; stack, stacking ensemble; p vote ≥ rad, p value for the null hypothesis that the mean performance when using solely radiomic features is at least as good as using both radiomic and topological features through a voting ensemble.

a Best mean performances with automated features.

### Lung tumor histology prediction (San Francisco/Palo Alto [SF/PA])

We considered three binary classification problems to evaluate TDA and compare it with radiomics-based lung tumor histology prediction: benign versus malignant, small cell versus non-small cell, and squamous versus ADC. For benign versus malignant, we see that adding topological features generally improves solely radiomic-features-based classification. In particular, using solely topological features already often outperforms radiomic features for classification. Nevertheless, the voting ensemble using both types of features consistently leads to the best performances. Topological features have the strongest (either strongly positive or strongly negative) correlation with radiomic features for scans with contrast, whereas they are less correlated for scans without contrast (Figures S3 and S4).

Next, for the small cell versus non-small cell classification problem, we observe that the topological features do not perform as well as radiomic features both for scans with and without added contrast. However, for images without added contrast, topological features do contribute to the final prediction model through a voting ensemble. Interestingly, both types of features appear to favor scans without contrast material for this particular classification problem. However, the performance differences between the base models with and without contrast are significantly higher for topological features than for radiomic features. It is noteworthy that there is little correlation between topological and radiomic features for scans with contrast, whereas some correlation can be found for scans without contrast (Figures S5 and S6). This may explain why the models using the different types of features are more on par for scans without contrast (Table 2).

Finally, for the ADC versus squamous cell carcinoma classification problem, we observe the highest performance increases when using topological features, most significantly for images with added contrast. Topological features perform both much better on their own as well as combined with radiomic features through concatenation or a voting ensemble. Interestingly, there is significantly more performance difference between with and without contrast material for topological features than for radiomic features. With a few exceptions, there is little correlation between the selected radiomic and topological features (Figures S7 and S8).

### Lung tumor malignancy prediction from radiologists’ assessment (LIDC)

The outcome here corresponds the continuous malignancy scores assigned by the radiologist for the 2,544 lung tumor nodules in the LIDC image collection, 807 scan with contrast material, and 1,737 without. Recall that these were made by the radiologist based on their visual assessments of a set of eight semantic features. For this outcome, the semantic features (which we averaged over different expert annotations) can thus, in some sense, be considered optimal for prediction. Naturally, there still remains variance in the outcome that is unexplained by solely the semantic features, e.g., due to averaging over the feature and outcome assessments made by different radiologists.

Considering the performances of the automated features, we observe that both with and without contrast material, radiomic features overall perform better than topological features. Thus, the radiomic features appear more applicable to mimic the manual predictions made by the radiologists. Nevertheless, we observe consistent improvements when combining radiomic with topological features through a voting ensemble.

### Lung tumor histology prediction from pathology ground truth (LIDC)

Finally, as discussed above, the manual predictions made by the radiologist are not always representative for the true lung tumor histology. We observe that while radiomic features were better at predicting the radiologist’s manual outcome annotations, topological features are actually better at predicting the accurate tumor diagnoses—at least on this smaller portion of the data for which they are available. Another interesting observation is that while the manually annotated semantic features perform best for images with contrast, they reach poor performance on images without contrast—unlike topological features. A possible explanation is that while the semantic features are valuable for predicting histology, their visual assessment may be more difficult without contrast.

## Discussion

In this article, we studied the use of topological features for various lung tumor histology classification and regression problems. In particular, we included a thorough overview for all principal prediction problems that might be considered from thoracic radiographic images of the lungs, further splitting our analysis into scans with and without added contrast material.

Our results consistently suggest that TDA provides a promising approach to lung tumor histology prediction from thoracic radiographic images, most notably for benign versus malignant and adeno versus squamous classification. Furthermore, as discussed in the supplemental information, we use a straightforward vectorization through summary statistics to obtain our topological features. Unavoidably, one loses information when transforming topological information into feature representations suitable for machine learning through such a (or any other) process. Therefore, a variety of complementary ways to learn through TDA exists. These already found successful machine-learning applications in medicine, in particular oncology, for example, to predict the survival prognosis of patients with lung cancer,^9^^,^^21^ predict the disease-free survival of patients with glioblastoma multiforme brain cancer,^10^ or analyze the heterogeneity in 3D thoracic CT images.^22^ Note that we do not claim our used topological feature extraction method to be superior to the methods described in these papers as we suggest investigating their applicability to the parallel problems that we studied in this article as further research. Thus—even though we already achieved encouraging results in this article—given the extensive manners to learn from TDA, much of its true potential for rapid diagnosis, intervention, customized treatment, and monitoring of patients with lung cancer is yet to be uncovered.

Furthermore, the contributions of our work are notably different from those in other recent studies on TDA for lung tumor histology prediction. For example,^21^ it focuses on survival analysis of non-SCLC while^22^ applying TDA to summarize textural information of lung ADCs. In contrast to this, our goal was to objectively study the potential of topological analysis for all primary lung tumor type classification problems, a study that, to the best of our knowledge, has not yet been performed. The fact that earlier work also demonstrates the effectiveness of TDA for (lung) tumor analysis further supports our findings on the Stanford unique data.

Beyond our main focus on the added effect of TDA for lung tumor histology prediction, our thorough performance evaluations led to extensive quantitative summarizations that are genuinely valuable. These include deeply interesting results open to further exploration, such as the different effects of contrast material or the performance differences between semantic, radiomic, and topological features when predicting the malignancy scores assigned by the radiologist versus when predicting the true tumor histology. For example, we found that while radiomic features may be more suitable for mimicking the radiologists’ malignancy annotations, topological features may actually be more suitable for predicting the true tumor histology.

## Experimental procedures

### Resource availability

#### Lead contact

Further information and requests for resources should be directed to the lead contact, Olivier Gevaert (ogevaert@stanford.edu).

#### Materials availability

No new materials were generated by this study.

### Quantitative image features extraction

#### Radiomic features

All images and masks were resampled to 1 × 1 × 1 mm^3^. Radiomic features were then extracted using PyRadiomics 1.0^24^ from the defined regions of interest. We selected 105 Image Biomarker Standardization Initiative (IBSI)11-compliant features across the following classes: first order statistics (19 features), shape-based (3D) (16 features), gray-level size zone matrix (GLSZM) (16 features), gray-level co-occurrence matrix (GLCM) (24 features), gray-level run length matrix (GLRLM) (16 features), gray-level size zone matrix (GLSZM) (16 features), neighboring gray tone difference matrix (NGTDM) (5 features), and gray-level dependence matrix (14 features).

#### Topological features

From each scan, we obtained different types of persistence diagrams. These diagrams, discussed in detail in the supplemental information, quantify topological holes in combinatorial objects termed simplicial complexes, which are higher-order generalizations of graphs that are constructed from the scan that grows (thus includes more simplices) with some time parameter t. This quantification is performed through birth-death pairs (b,d), which characterize a topological hole that appeared (was born) at time t=b∈R and that (possibly never) disappeared (died), a time t=d∈R∪{∞}. Topological holes in the lesion, and thus persistence diagrams, can be distinguished by their dimension: connected components (dimension 0), cycles (dimension 1), and voids (dimension 2). These persistence diagrams can furthermore be distinguished by the object through which they capture topological information from the tumor. We considered five such objects, namely the lesion pixels (raw and negated), the lesion pixels with boundary box pixels (raw and negated), and a point cloud representing the lesion surface. Hence, from each scan, we obtained 5 × 3 = 15 persistence diagrams: 3 dimensions of holes for each of the 5 objects. Figure 2 illustrates 3 × 3 examples of such diagrams and the objects they are computed from. Finally, we obtained summary statistics from these diagrams, as detailed below, resulting in a vector of 290 topological features per scan.

### Supervised machine-learning modeling

For each of our machine-learning models (discussed hereafter), we used the same feature preprocessing, consisting of the following steps: (1) missing values (which rarely occurred when a persistence diagram was empty) were imputed with the mean; (2) features were minimum-maximum (min-max) scaled to [0, 1]; (3) features were binned into five partitions of equal length, which serves the following feature selection method that takes discretized features; and (4) to maintain a similar number of radiomic and topological features and to reduce the effects of overfitting, we used a feature selection procedure based on minimum redundancy maximum relevance (mRMR) to select 10 features for the final prediction model.^25^ We then combined this preprocessing pipeline with each one of six commonly applied classification or regression models (depending on the outcome), namely logistic/linear regression (LR), random forest classification/regression (RF), k -nearest neighbor classification/regression (KNN), support vector machine/regressor (SV), Gaussian naive Bayes classification/Bayesian regression (BAY), and extreme gradient-boosted trees classification/regression (XGB). Note that the same abbreviation may represent a different machine-learning model depending on whether classification or regression is considered and that most abbreviations are only used in Tables S1–S10. Finally, to compare radiomic and topological features, we used various types of feature and model combinations to evaluate the histology prediction models, namely training on radiomic features only (rad); training on topological features only (top); training on the concatenated features (concat); a soft-voting ensemble using models trained on the radiomic and topological features separately (vote); and a stacking ensemble, which is—unlike the voting ensemble that combines the output directly—retrained on the probabilistic output of the models that where trained on both feature types separately (stack). The stacking estimator was equal to the base estimators, e.g., if the base estimators were logistic regression models then so was the final estimator. Note that there were insufficient scans with contrast of squamous tumors in the SF/PA cohort to evaluate a stacking classifier through cross-validation.

**Figure 3:**
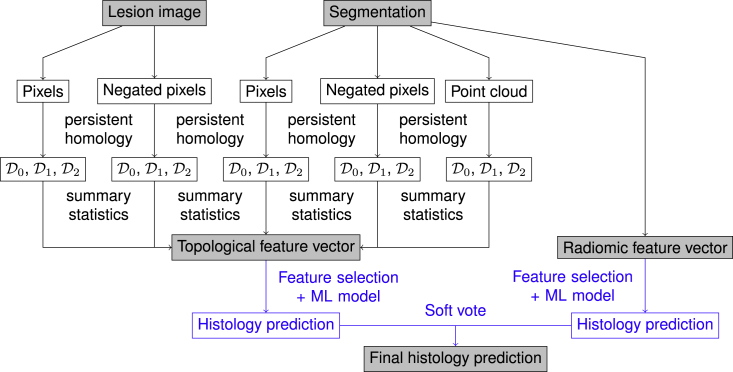
Schematic overview of the machine-learning pipeline we present in this article The part shown in blue may vary depending on which type of features we use or how they are combined. The example shown is for a voting ensemble that uses both types of features.

Figure 3 shows a schematic overview of the machine-learning pipeline we applied to evaluate and compare topological and radiomic features for lung tumor histology prediction.

### Model evaluation

To evaluate the models and thus the features, we used 10 repeats of 5-fold cross-validation to measure the performance of the classification (receiver operating characteristic [ROC] area under the curve [AUC]) and regression ($r^{2}$ coefficient of determination) models. Per classification/regression problem, the folds were kept the same over all model evaluations. For the classification problems on the SF/PA data, we used stratified sampling to obtain the folds. This is especially important given the small sample sizes, e.g., to ensure that each fold contains examples of both classes. For the regression problems on the LIDC data, we used standard random sampling. However, this sampling was conducted on the patient level rather than the nodule level, ensuring that different nodules from the same patient were within the same fold. For the classification problems on the LIDC data, we also used stratified sampling. For this, we constructed a class variable indicating whether a patient had a benign tumor nodule (only rarely did a patient have both benign and malignant nodules) as to again be able to perform the sampling on the patient level. Note that this class variable was not used for the final outcome as the lung tumor histology predictions were made on the nodule level.

## Data Availability

Data to replicate the results summarized in this paper are available from GitHub:robinvndaele.^23^ This includes persistence diagrams, features, metadata, and outcomes for both the SF/PA and LIDC data. Original scans and masks for the SF/PA cohort are excluded from this repository and are not permitted to be shared. Original LIDC scans and masks are publicly available from TCIA: https://doi.org/10.7937/K9/TCIA.2015.LO9QL9SX. All code for this project is available on GitHub:robinvndaele.^23^

## Supplemental information

Document S1. Supplemental experimental procedures, Figures S1–S12, and Tables S1–S10

Document S2. Article plus supplemental information
