# Supplementary material for: Topological data analysis of thoracic radiographic images shows improved radiomics-based lung tumor histology prediction
Source: Patterns (N Y). 2022 Dec 12;4(1):100657. doi: 10.1016/j.patter.2022.100657 (PMC9868648; doi:10.1016/j.patter.2022.100657)
Supplement: Document S1. Supplemental experimental procedures, Figures S1–S12, and Tables S1–S10 [file mmc1.pdf]

Patterns, Volume *4*

Supplemental information

Topological data analysis of thoracic radiographic images shows improved radiomics-based lung tumor histology prediction

Robin Vandaele, Pritam Mukherjee, Heather Marie Selby, Rajesh Pravin Shah, and Olivier Gevaert

SUPPLEMENTAL INFORMATION

Supplemental experimental procedures

Persistent homology. *Persistent homology* is unarguably the most studied and applied method in topological data analysis (TDA). Its roots are in the field of *algebraic topology*[S1], where it has been developed to quantify changes in topological *holes* across a *filtration*, i.e., an ordered sequence of *simplicial complexes*

$$\mathcal{F} = K_0 \subseteq K_1 \subseteq \ldots \subseteq K_N = K$$

of an initial complex K. A simplicial complex K can be seen as a generalization of a graph, that apart from nodes (0-simplices) and edges (1-simplices), also includes *higher-dimensional simplices* such as triangles (2-simplices), tetrahedra (3-simplices), ..., with the constraint that if K contains a simplex σ, every simplex $\sigma' \subseteq \sigma$ must also be contained in K. Figure S1a illustrates an example of such a filtration.

The topological holes that are quantified through persistent homology, are characterized by their dimension as follows.

- 0-dimensional holes correspond to gaps between connected components.
- 1-dimensional holes correspond to the inside of a loop, such as the inside of a ring or the handle of a coffee mug.
- 2-dimensional holes correspond to voids, such as the inside of a balloon.
- In general, a k-dimensional hole corresponds to the inside of a k-sphere. They can only occur in a space of at least dimension $k+1$. For $k \geq 3$, these holes become difficult to visualize. These holes are also not used in this paper, since they never occur in the mathematical objects from which we compute persistent homology.

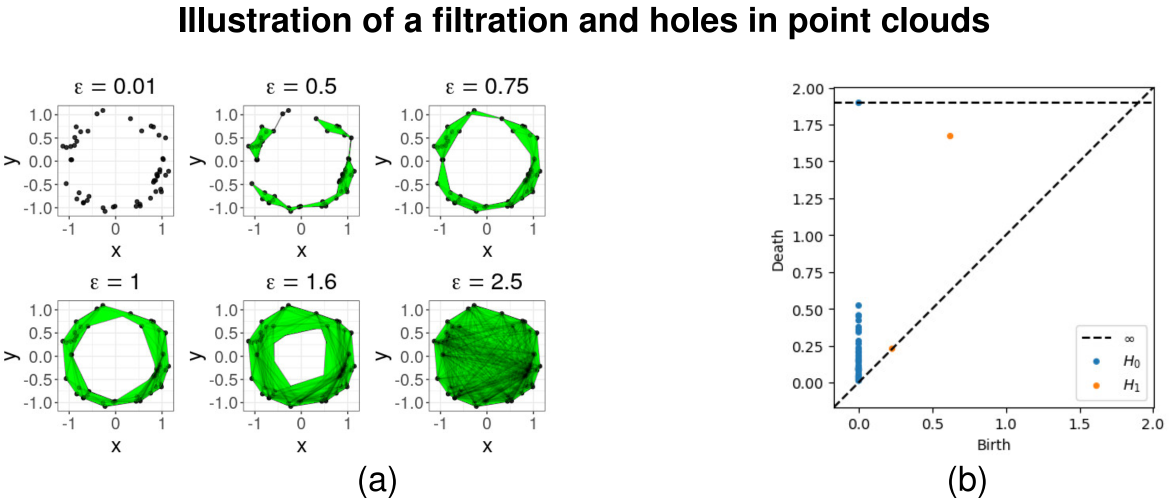

FIGURE S1. (A) An example of various simplicial complexes in a filtration constructed from a point cloud dataset. Here, the filtration equals the Vietoris-Rips filtration, parameterized by a time (distance) parameter ϵ. At time ϵ, all simplices with at most three nodes and a diameter—that is, the maximal pairwise distance between the nodes of the simplex—of at most ϵ are included in the complex. (B) The two corresponding persistence diagrams, one for each considered dimension of hole, plotted on top of each other. Note that this example does not relate to our tumor imaging data: it is mainly used as an interpretable illustration of what persistent homology is able to quantify. The two most elevated points quantify the connected component (H0) and cycle (H1) in the underlying circular model of the data, as explained below. Observe that for our application however, we also aim to learn from topological information on a smaller scale, not only from the most prominent holes.

The number of k-dimensional holes of a simplicial complex is denoted by the *Betti-number* β_k. In particular, β_0 denotes the number of connected components.

Given a simplicial complex K, and a real-valued function f defined on all simplices in K, a filtration can generally be written in the form of a *sublevel filtration*

$$\mathcal{F} = \{\sigma \in K : f(\sigma) \leq t\}_{t\in\mathbb{R}},$$

parameterized by a *time* parameter t. Note that the term 'time' is to be loosely interpreted, as the parameter can also capture another unit such as distance (Figure S1) or pixel intensity (Figure S2). In practice, i.e., when dealing with finite data, simplicial complexes in $\mathcal{F}$ change for only finitely many values $t_0, \ldots, t_N \in \mathbb{R}$. E.g., the filtration in Figure S1a equals the *Vietoris-Rips* filtration, where K contains all subsets of a given metric space, i.e., a point cloud dataset, and f maps each subset to its diameter. In this case the dataset is 3-dimensional, and hence, no holes of dimension 3 or higher can occur. Thus, we do not include simplices that are higher-dimensional, i.e., include more points, than triangles. The Vietoris-Rips filtration is used to obtain topological information from the point cloud modeling the tumors surfaces (Figures 2d and 2g).

A filtration can also be obtained directly from the CT scan image pixels of a tumor, quantifying the radiodensity of the tissue. Note that such scan can be regarded as a 3D array of pixels, of which an example slice restricted to the tumor pixels is shown in Figure 2b. By connecting neighboring pixels, that is, pixels that are horizontally, vertically, or diagonally adjacent to each other, in a particular manner, we obtain a three-dimensional complex K that is known as *Fruedenthal's triangulation*[1S4]. The original image can now be regarded as a real-valued function f defined on the 0-simplices in K, where for a 0-simplex (pixel) p, $f(p)$ denotes the intensity of p in the original image. Through this function, we can define a sublevel filtration directly from the pixel values of the original image as

$$\mathcal{F} = \left\{\sigma \in K : f(\sigma) := \max_{p\in\sigma}(p) \leq t\right\}_{t\in\mathbb{R}}.$$

Intuitively, the complex at time t is induced by all pixels with intensity at most t, and their neighboring relations. The resulting filtration for the image in Figure 2b is shown in Figure S2a. By being inherently 3-dimensional, only up to 2-dimensional holes (voids) can occur in the filtration. The range of our filtration is determined by the minimum and maximum pixel value, and thus is chosen in a parameter-free manner.

For both types of filtrations, whether derived from a point cloud or image pixels, persistent homology tracks the *birth* (b) and *death* (d) of these holes across the filtration. The obtained tuples (b, d) are then commonly visualized by means of a *persistence diagram*, one for each dimension of hole. E.g., Figure S1b shows the persistence diagrams of the Vietoris-Rips filtration in Figure S1a. In this filtration, every point defines the birth of a connected component at time $\epsilon = 0$. These correspond to the blue points in Figure S1b (H0). By connecting more and more distant points by edges, and 'filling in' the resulting triangles, we see that many connected components die (they merge with others). Eventually a 1-dimensional hole (a circle) is formed by the complex, which persists for a relatively long time in the filtration, and finally gets filled in and thus dies. This circle is marked by the highly elevated orange point (H1) in Figure S1b. For the filtration in Figure S2a (of which the persistence diagrams are shown in Figure 2e), the darkest pixels correspond to connected components that are born first. When brighter pixels are consecutively added during the filtration, they may either give rise to new connected components (which are then born as well), or immediately connect to darker pixels which were already present. In the latter case, they may also merge two previously disconnected components, resulting in the death of a connected component. Although 1- and 2-dimensional holes may also be born and die (or even persist indefinitely) during this filtration, this is less apparent from Figure S2a. This becomes more intuitive from Figure S2b.

[1]Another popular type of complex for image data is the *cubical complex*[S2]. It can be straightforwardly defined on a 3D image by connecting all neighboring pixels that are either horizontally or vertically adjacent to each other. The connected points then form the edges, squares, and cubes of which the complex is composed. Cubical complexes are thus similar to simplicial complexes, but with hypercubes instead of hypertriangles. Using cubical complexes to compute persistent homology from images is standard practice[S3]. We opted for the Fruedenthal's triangulation however because of our familiarity with the Dionysus library in Python in which it was readily available. By no means however do we claim Fruedenthal's triangulation to be superior over cubical complexes for this application. We do not expect significant differences when applying the different types of complexes for our application, as they are meant to capture similar topological information.

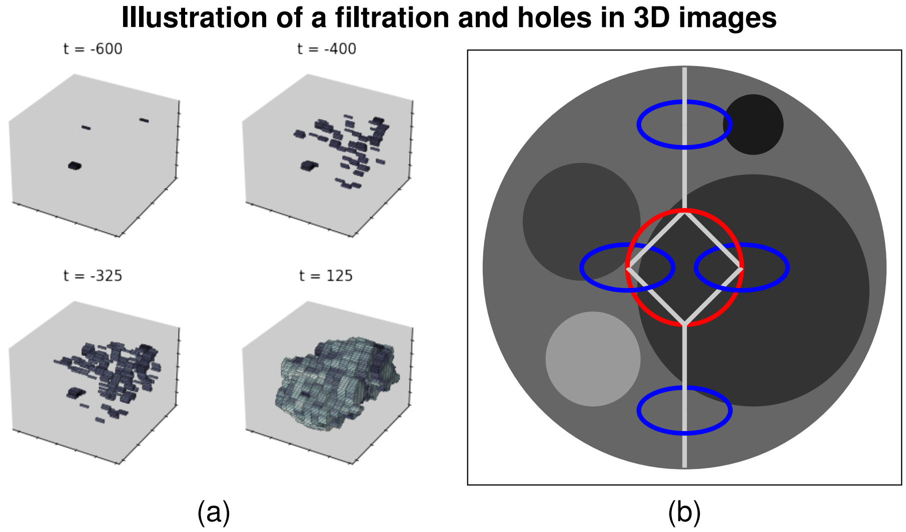

FIGURE S2. (A) An example of various simplicial complexes in a filtration constructed from the segmented 3D tumor image of which one 2D slice is shown in Figure 2a. Here, the filtration equals the sublevel filtration defined by the function f mapping each pixel (0-simplex) to its value in the original CT scan image. Darkest pixels are included first. By increasing the time parameter t, increasingly brighter pixels are included in the complex as well. 1-, 2-, and 3-simplices are induced by the neighboring relationships between pixels, and not shown in this illustration. (B) A simplified artificial illustration of how various holes may occur in a 3D grayscale image. The illustration should be imagined as representing the restrictions of the holes to a 2D-slice of a 3D-image. The largest sphere represents a lesion, the smaller enclosed spheres tissue components of varying radiodensity within the lesion, and the white lines blood vessels that would appear brighter on scans with contrast material. In a sublevel filtration, the darkest pixels are included first. The enclosed spheres that are darker than the surrounding pixels will thus be included first as separate components, until they merge when the brighter surrounding pixels are included as well. These components will thus be detected in the 0-dimensional persistence diagram of the sublevel filtration, where their birth time b represents the radiodensity of the tissue component, and their death time d represents the radiodensity of the surrounding tissue. Note that these components would similarly be detected as voids when brighter pixels would have been included first, i.e., through the 2-dimensional persistence diagram of the superlevel filtration. An analogous explanation holds for enclosed spheres that are brighter than the surrounding pixels. In case of 1-dimensional persistence, the holes surrounding the blood vessels (blue circles) will be discovered through the sublevel filtration, as the vessels themselves will be excluded from the filtration until the brightest pixels are included at the end of the filtration. The holes defined by the vessels themselves (red circle) will be discovered through the superlevel filtration, as the interior tissue would be excluded until the darker pixels are included. Unlike radiomic features which may also capture properties such as tissue homogeneity and vascularity, persistent homology quantifies topological properties over the entire (color) scale of the data. Our topological features summarize their distribution across this scale.

Learning from persistence diagrams. One of the main original ideas behind persistent homology and persistence is that holes persisting for a long time–which correspond to 'highly elevated' points in their respective persistence diagram—-represent significant features of the underlying topology (hence the name 'persistence'). E.g., this in Figure S2a the single highly elevated point for connected components (H0) represents that the underlying topological model is connected, and the single highly elevated point for loops (H1) that the underlying topological model contains a cycle.

More recently it has been shown that the entire distribution of points on a persistence diagram may play a significant role in characterizing the data[S5–S7]. This is exactly the power of persistent homology for machine learning: it quantifies all of the finest up to the coarsest of topological information in data. A prominently persisting void in the tumor surface may quantify the tumor eccentricity, whereas shorter persisting cycles or voids may quantify handles or lobes on, or thus, textural properties of the tumor surface. In the CT scan, the occurrence of many isolated connected components or voids may indicate a more 'spongy' textured tumor. Especially on images with contrast in which these vessels appear brighter, loops may either surround blood vessels or mark circular structures in the vessel trajectories themselves, as shown in Figure S2b. Our topological features then summarize both the occurrence, sizes, quantities, and the distribution of all these types of structural properties. Note that providing a full list of how topological holes may manifest in tumors is beyond our expertise as well as the scope of this paper, and should be ideally analyzed in future work.

As multisets, persistence diagrams cannot be straightforwardly incorporated into many machine learning models. Various methods have been developed to overcome this issue, as we summarize below.

- Vectorized features of a fixed size can be computed from the persistence diagrams. These can be summary statistics, such as the number of points, or various moments (raw, central, standardized) obtained from their lifetimes. This is the approach we used in this paper. Other examples include Betti curves[S8], or discretizations of the persistence diagram density such as persistent images[S9].
- Various kernel methods have been developed for learning from persistence diagrams, such as the persistence Fisher kernel[S10] and the persistence weighted Gaussian kernel[S11].
- Deep learning variants are recently getting more attention[S12–S15]. These are designed to learn a task optimal representation of the persistence diagrams at hand.

For a good overview of more general vectorized and kernelized methods that are designed to learn from persistence diagrams, compatible with the SCIKIT-LEARN library in Python, we recommend[S16].

In this paper, we focus on vectorized features through summary statistics. This allows us to include interpretable feature selection, and lacks the need of choosing hyperparameters, which would be difficult to optimize for our small data sizes. In this way, we are able to construct and evaluate the exact same machine learning pipeline to compare both types of features, i.e., radiomic and topological. Nevertheless, we do not claim this type of feature extraction method to be superior to any of the other methods described above.

Filtrations summarizing topological information in lung lesions. From each scan, we obtained the following five to compute topological information from through persistent homology.

(1) The sublevel filtration obtained from the raw pixel values, restricted to the segmented lesion (see also Figures 2b and 2e).
(2) Same as above, but with negated pixel values.
(3) The sublevel filtration obtained from the raw pixel values, restricted to the boundary box of the segmented lesion. The boundary box is determined by the minimal and maximal (x, y, z) coordinates of the segmentation in the original 3D image scan, and includes topological information of lung tissue surrounding the lesion (see also Figures 2c and 2f).
(4) Same as above, but with negated pixel values.
(5) From the given binary lesion segmentation, we first obtained its surface mesh using the marching cubes algorithm[S17]. Consecutively, we computed the Vietoris-Rips filtration on the vertices of the mesh, thus a point cloud in the Euclidean space $\mathbb{R}^3$ (see also Figure 2d). For computational purposes, the persistence diagrams are approximated through the method described in[S18] using 1000 landmark points (Figure 2g). These landmarks are derived using a furthest-point sampling method, where the next furthest point from all current landmarks is iteratively added to the set of landmarks, which is randomly initialized with one landmark. This procedure provides an even distribution of the selected landmarks. It is however sensitive to outliers, but we do not experience these to affect our particular type of data.

Persistent homology computation. Computing persistence diagrams was performed in Python using DIONYSUS (`https://pypi.org/project/dionysus/`) for image pixels, and RIPSER[S19] for point clouds.

Topological features for lung tumor histology prediction, 20 summary statistics of the persistence diagrams were collected into a topological feature vector. First, each birth-death pair (b, d) was transformed

to a *lifespan* $d-b$ and a *midlife* $\frac{b+d}{2}$ in $\mathbb{R}_{>0} \cup \{\infty\}$. These can be interpreted as the prominence, respectively, the location, of a point in the diagram. From each diagram we computed the following statistics.

(1) The minimal birth-time.
(2) The number of infinite lifespans.
(3) The number of finite lifespans.
(4-19) The mean, standard deviation, skewness, kurtosis, first quartile, median, third quartile, and interquartile range of the finite lifespans and finite midlifes.
(20) The entropy of the finite lifespans[S20].

Per scan, this thus resulted in a topological feature vector of size $5 \times 3 \times 20 = 300$. However, the number of infinite lifetimes is always the same for the six diagrams obtained from the images with boundary box pixels, as well as for the three diagrams obtained from the point clouds, which all have one 0-dimensional hole and no higher-dimensional holes that persist indefinitely. Similarly, the lowest birth-time in a diagram of the 0-dimensional holes in a point cloud is always 0. These features were thus omitted, resulting in a final vector of $300 - 6 - 3 - 1 = 290$ topological features per scan.

We observed that in a few cases there were diagrams without any point (b, d) for which $d < \infty$. The summary statistics from the finite lifespans and midlifes are then not straightforwardly defined. In case of the lifespans, we reasoned that any hole that would have been born, died immediately. The statistics for the finite lifespans were then always defined to be 0, analogous to how they would be defined for a random variable that always evaluates to 0. However, an analogous interpretation for the finite midlifes is more difficult. We therefore treated their summary statistics as missing values.

Note that there may be overlapping information in the persistence diagrams obtained from the filtration of the original and negated image, albeit in different dimensions. For example, voids in one filtration may correspond to connected components in the other (Figure S2b). Therefore, an effective feature selection method as we used in our pipeline, is of crucial importance.

Hyperparameters and settings. For all considered models, we used their standard settings from the Python libraries SCIKIT-LEARN and XGBOOST, apart from changing the output to be probabilistic if needed, i.e., to obtain ROC AUC scores.

References

[S1] A. Hatcher, Algebraic topology, Cambridge University Press, 2002.
[S2] T. Kaczynski, K. M. Mischaikow, M. Mrozek, Computational homology, Vol. 3, Springer, 2004.
[S3] D. Ziou, M. Allili, Generating cubical complexes from image data and computation of the euler number, Pattern Recognition 35 (12) (2002) 2833–2839.
[S4] C. Dang, Triangulations and simplicial methods, Vol. 421, Springer Science & Business Media, 2012.
[S5] O. Dunaeva, H. Edelsbrunner, A. Lukyanov, M. Machin, D. Malkova, R. Kuvaev, S. Kashin, The classification of endoscopy images with persistent homology, Pattern Recognition Letters 83 (2016) 13–22.
[S6] N. Kadoya, S. Tanaka, T. Kajikawa, S. Tanabe, K. Abe, Y. Nakajima, T. Yamamoto, N. Takahashi, K. Takeda, S. Dobashi, et al., Homology-based radiomic features for prediction of the prognosis of lung cancer based on ct-based radiomics, Medical Physics 47 (5) (2020) 2197–2205.
[S7] M. Wang, Z. Cang, G.-W. Wei, A topology-based network tree for the prediction of protein–protein binding affinity changes following mutation, Nature Machine Intelligence 2 (2) (2020) 116–123.
[S8] Y. Umeda, Time series classification via topological data analysis, Information and Media Technologies 12 (2017) 228–239.
[S9] H. Adams, T. Emerson, M. Kirby, R. Neville, C. Peterson, P. Shipman, S. Chepushtanova, E. Hanson, F. Motta, L. Ziegelmeier, Persistence images: A stable vector representation of persistent homology, Journal of Machine Learning Research 18 (2017).
[S10] T. Le, M. Yamada, Persistence fisher kernel: A riemannian manifold kernel for persistence diagrams, arXiv preprint arXiv:1802.03569 (2018).
[S11] G. Kusano, Y. Hiraoka, K. Fukumizu, Persistence weighted gaussian kernel for topological data analysis, in: International Conference on Machine Learning, PMLR, 2016, pp. 2004–2013.
[S12] C. Hofer, R. Kwitt, M. Niethammer, A. Uhl, Deep learning with topological signatures, in: I. Guyon, U. V. Luxburg, S. Bengio, H. Wallach, R. Fergus, S. Vishwanathan, R. Garnett (Eds.), Advances in Neural Information Processing Systems, Vol. 30, Curran Associates, Inc., 2017.
[S13] M. Zaheer, S. Kottur, S. Ravanbakhsh, B. Poczos, R. R. Salakhutdinov, A. J. Smola, Deep sets, in: I. Guyon, U. V. Luxburg, S. Bengio, H. Wallach, R. Fergus, S. Vishwanathan, R. Garnett (Eds.), Advances in Neural Information Processing Systems, Vol. 30, Curran Associates, Inc., 2017.
[S14] M. Carrière, F. Chazal, Y. Ike, T. Lacombe, M. Royer, Y. Umeda, Perslay: a neural network layer for persistence diagrams and new graph topological signatures, in: International Conference on Artificial Intelligence and Statistics, PMLR, 2020, pp. 2786–2796.

[S15] M. Carriere, F. Chazal, M. Glisse, Y. Ike, H. Kannan, Y. Umeda, Optimizing persistent homology based functions, in: International Conference on Machine Learning, PMLR, 2021, pp. 1294–1303.

[S16] The GUDHI Project, GUDHI User and Reference Manual, 3.4.1 Edition, GUDHI Editorial Board, 2021. URL `https://gudhi.inria.fr/doc/3.4.1/`

[S17] W. E. Lorensen, H. E. Cline, Marching cubes: A high resolution 3d surface construction algorithm, ACM siggraph computer graphics 21 (4) (1987) 163–169.

[S18] N. Cavanna, M. Jahanseir, D. Sheehy, A geometric perspective on sparse filtrations, arXiv preprint arXiv:1506.03797 (2015).

[S19] C. Tralie, N. Saul, R. Bar-On, Ripser.py: A lean persistent homology library for python, The Journal of Open Source Software 3 (29) (2018) 925.

[S20] E. Merelli, M. Rucco, P. Sloot, L. Tesei, Topological characterization of complex systems: Using persistent entropy, Entropy 17 (10) (2015) 6872–6892.

Supplemental items

Performances for benign vs. malignant (classification, SF/PA, with contrast)

model	rad	top	concat	vote	stack
LR	86.7 ± 8.7	87.5 ± 10.9	87.2 ± 10.7	$\mathbf{88.9 \pm 9.2}$	88.9 ± 9.2
RF	85.7 ± 12.0	87.9 ± 11.0	87.5 ± 11.6	$\mathbf{88.8 \pm 11.4}$	86.6 ± 11.6
KNN	83.9 ± 11.5	87.2 ± 10.6	87.8 ± 10.0	$\mathbf{87.9 \pm 10.6}$	83.7 ± 10.5
SV	84.4 ± 9.9	85.0 ± 11.6	84.4 ± 11.7	$\mathbf{88.0 \pm 9.5}$	87.0 ± 9.6
BAY	84.9 ± 10.3	85.8 ± 11.9	86.7 ± 12.1	$\mathbf{87.6 \pm 9.5}$	87.2 ± 9.8
XGB	81.9 ± 12.8	$\mathbf{87.6 \pm 12.3}$	86.6 ± 13.4	86.6 ± 11.6	81.5 ± 14.6
mean	84.6 ± 11.0	86.8 ± 11.5	86.7 ± 11.7	$\mathbf{87.9 \pm 10.4}$	85.8 ± 11.3

Table S1. ROC AUC performances in % with standard deviations for *benign vs. malignant* classification of lung tumor CT scan images *with added contrast*, using radiomic features (*rad*) and topological features (*top*), as well as for three models combining both: through concatenation (*concat*), soft voting (*vote*), and stacking. Each scores is averaged over 50 models, obtained through 10-repeated stratified samplings in 5 folds. Best scores are marked in bold.

Performances for benign vs. malignant (classification, SF/PA, without contrast)

model	rad	top	concat	vote	stack
LR	75.6 ± 10.7	77.7 ± 10.0	79.0 ± 9.1	$\mathbf{80.2 \pm 9.6}$	79.7 ± 9.5
RF	72.7 ± 8.8	76.3 ± 8.9	75.9 ± 9.8	$\mathbf{77.9 \pm 9.3}$	69.5 ± 9.7
KNN	72.3 ± 10.3	75.4 ± 9.8	75.5 ± 9.6	$\mathbf{77.8 \pm 9.7}$	72.8 ± 10.1
SV	74.5 ± 10.8	76.2 ± 10.1	78.1 ± 9.4	$\mathbf{78.9 \pm 9.9}$	78.5 ± 10.1
BAY	$\mathbf{78.7 \pm 9.7}$	74.2 ± 10.6	74.7 ± 10.4	77.9 ± 9.1	76.5 ± 9.8
XGB	70.0 ± 10.2	74.7 ± 11.0	75.5 ± 11.0	$\mathbf{76.5 \pm 9.8}$	65.6 ± 12.7
mean	74.0 ± 10.5	75.7 ± 10.1	76.5 ± 10.0	$\mathbf{78.2 \pm 9.6}$	73.8 ± 11.5

Table S2. ROC AUC performances in % with standard deviations for *benign vs. malignant* classification of lung tumor CT scan images *without added contrast*, using radiomic features (*rad*) and topological features (*top*), as well as for three models combining both: through concatenation (*concat*), soft voting (*vote*), and stacking. Each scores is averaged over 50 models, obtained through 10-repeated stratified samplings in 5 folds. Best scores are marked in bold.

Feature correlation for benign vs. malignant (classification, SF/PA, with contrast)

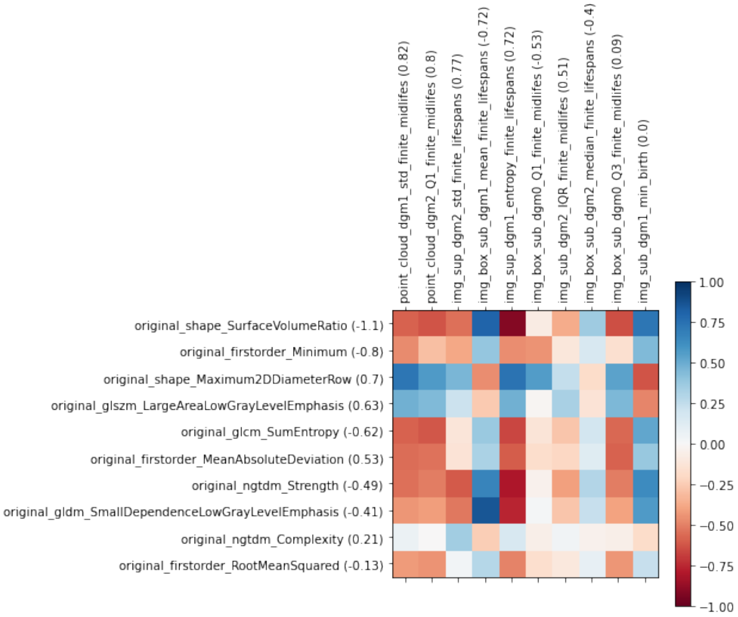

FIGURE S3. Correlation between the radiomic and topological features selected by the LR models for benign vs. malignant classification with contrast (SF/PA).

Feature correlation for benign vs. malignant (classification, SF/PA, without contrast)

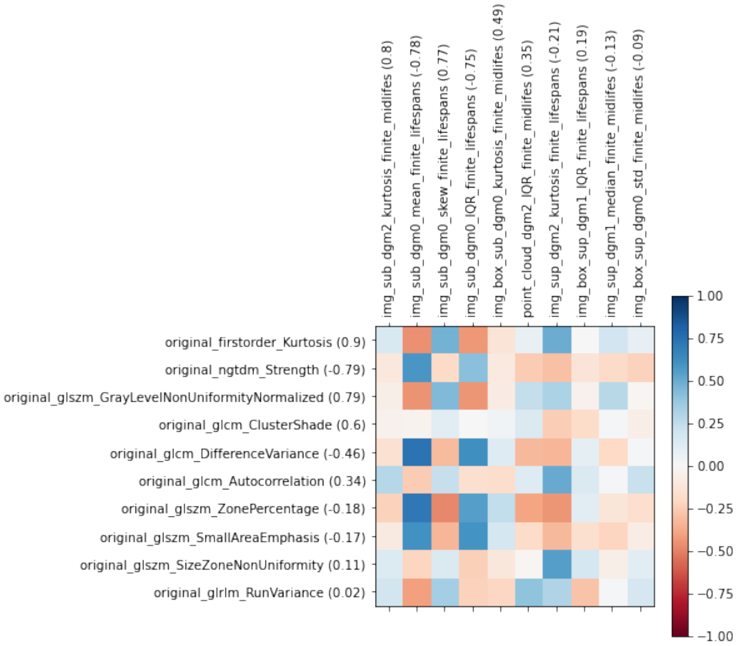

FIGURE S4. Correlation between the radiomic and topological features selected by the LR models for benign vs. malignant classification without contrast (SF/PA).

Performances for small-cell vs. non-small cell (classification, SF/PA, with contrast)

model	rad	top	concat	vote	stack
LR	**79.8 ± 18.1**	62.6 ± 19.7	62.1 ± 20.9	75.7 ± 19.6	74.4 ± 21.1
RF	**78.4 ± 18.8**	63.2 ± 19.6	73.8 ± 17.7	76.0 ± 19.4	77.2 ± 20.0
KNN	76.8 ± 17.3	64.2 ± 19.5	66.8 ± 19.3	**77.2 ± 18.9**	74.8 ± 19.1
SV	**79.8 ± 18.1**	62.6 ± 18.4	60.8 ± 20.8	75.1 ± 19.5	69.1 ± 25.7
BAY	**73.7 ± 20.6**	62.5 ± 18.4	61.5 ± 18.4	72.8 ± 19.10	64.9 ± 26.5
XGB	**76.4 ± 17.2**	61.4 ± 22.0	71.8 ± 20.6	73.3 ± 17.1	69.7 ± 21.3
mean	**77.5 ± 18.5**	62.7 ± 19.7	66.1 ± 20.3	75.0 ± 19.0	71.7 ± 22.8

Table S3. ROC AUC performances in % with standard deviations for *small cell vs. non-small cell* classification of lung tumor CT scan images *with added contrast*, using radiomic features (*rad*) and topological features (*top*), as well as for three models combining both: through concatenation (*concat*), soft voting (*vote*), and stacking. Each scores is averaged over 50 models, obtained through 10-repeated stratified samplings in 5 folds. Best scores are marked in bold.

Performances for small-cell vs. non-small cell (classification, SF/PA, without contrast)

model	rad	top	concat	vote	stack
LR	80.4 ± 21.5	79.8 ± 19.2	81.6 ± 20.0	**82.6 ± 19.7**	81.6 ± 20.2
RF	83.8 ± 18.9	81.7 ± 19.3	84.4 ± 19.4	**86.8 ± 17.3**	83.8 ± 17.6
KNN	79.1 ± 18.4	78.3 ± 19.0	81.3 ± 18.6	**83.6 ± 19.3**	71.8 ± 24.0
SV	77.9 ± 21.4	77.3 ± 20.2	80.8 ± 21.7	**81.4 ± 20.8**	66.1 ± 29.9
BAY	82.3 ± 19.6	75.9 ± 24.2	77.2 ± 21.2	82.1 ± 19.2	**82.9 ± 19.8**
XGB	80.2 ± 20.1	78.8 ± 22.3	80.3 ± 20.2	**84.1 ± 17.5**	69.5 ± 27.8
mean	80.6 ± 20.1	78.6 ± 20.9	80.9 ± 20.3	**83.4 ± 19.1**	75.9 ± 24.7

Table S4. ROC AUC performances in % with standard deviations for *small cell vs. non-small cell* classification of lung tumor CT scan images *without added contrast*, using radiomic features (*rad*) and topological features (*top*), as well as for three models combining both: through concatenation (*concat*), soft voting (*vote*), and stacking. Each scores is averaged over 50 models, obtained through 10-repeated stratified samplings in 5 folds. Best scores are marked in bold.

Feature correlation for small-cell vs. non-small cell (classification, SF/PA, with contrast)

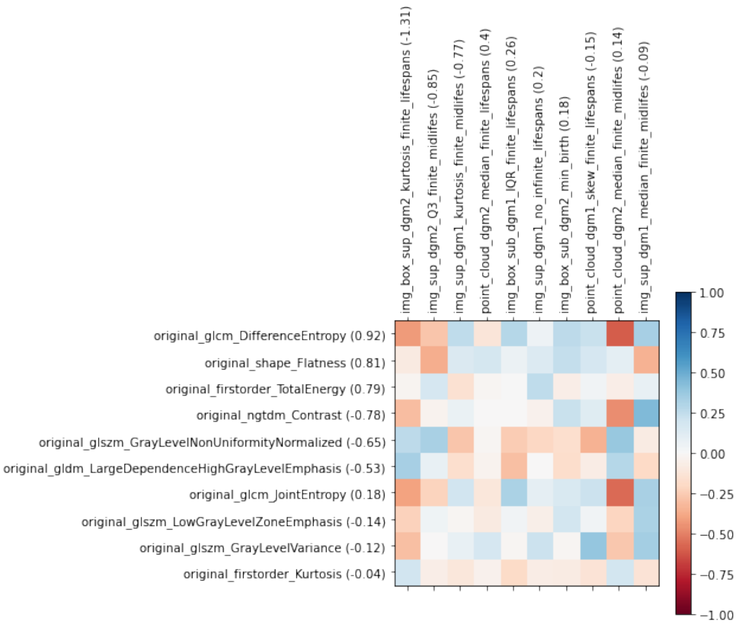

Figure S5. Correlation between the radiomic and topological features selected by the LR models for small-cell vs. non-small cell classification with contrast (SF/PA).

Feature correlation for small-cell vs. non-small cell (classification, SF/PA, without contrast)

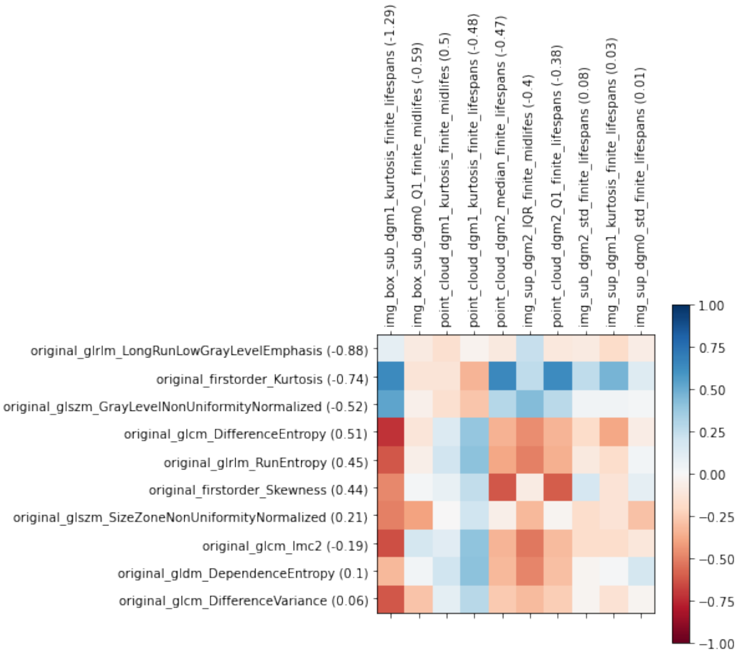

Figure S6. Correlation between the radiomic and topological features selected by the LR models for small-cell vs. non-small cell classification without contrast (SF/PA).

Performances for adeno vs. squamous (classification, SF/PA, with contrast)

model	rad	top	concat	vote	stack
LR	80.2 ± 28.0	$\mathbf{97.3 \pm 10.7}$	$\mathbf{97.3 \pm 10.7}$	96.3 ± 16.1	-
RF	66.8 ± 37.6	$\mathbf{98.3 \pm 8.3}$	$\mathbf{98.3 \pm 8.3}$	91.0 ± 21.9	-
KNN	54.5 ± 25.9	$\mathbf{89.7 \pm 21.6}$	87.2 ± 21.2	86.0 ± 25.1	-
SV	68.5 ± 33.2	94.4 ± 18.4	$\mathbf{97.0 \pm 11.9}$	96.7 ± 15.3	-
BAY	65.5 ± 32.9	70.8 ± 27.1	65.5 ± 29.5	$\mathbf{73.0 \pm 34.2}$	-
XGB	67.2 ± 37.0	96.8 ± 11.2	95.2 ± 14.3	86.8 ± 23.5	-
mean	67.2 ± 33.6	$\mathbf{91.2 \pm 20.0}$	90.1 ± 21.1	88.3 ± 24.8	-

Table S5. ROC AUC performances in % with standard deviations for *adeno vs. squamous* classification of lung tumor CT scan images *with added contrast*, using radiomic features (*rad*) and topological features (*top*), as well as for three models combining both: through concatenation (*concat*), soft voting (*vote*), and stacking. Each scores is averaged over 50 models, obtained through 10-repeated stratified samplings in 5 folds. Best scores are marked in bold. Note that there were insufficient examples of squamous tumors to train a stacking classifier.

Performances for adeno vs. squamous (classification, SF/PA, without contrast)

model	rad	top	concat	vote	stack
LR	62.9 ± 22.4	71.9 ± 16.9	70.7 ± 17.8	$\mathbf{72.1 \pm 18.6}$	69.9 ± 17.9
RF	63.8 ± 25.5	67.8 ± 13.9	66.8 ± 17.6	$\mathbf{70.6 \pm 15.8}$	60.6 ± 23.1
KNN	64.3 ± 19.4	71.0 ± 15.4	69.8 ± 16.3	$\mathbf{74.2 \pm 16.4}$	67.9 ± 18.0
SV	65.9 ± 22.0	71.4 ± 17.3	$\mathbf{71.7 \pm 16.9}$	71.6 ± 17.7	68.3 ± 19.6
BAY	70.0 ± 22.3	69.7 ± 16.9	69.5 ± 16.3	$\mathbf{75.0 \pm 21.7}$	72.2 ± 21.6
XGB	58.8 ± 24.5	$\mathbf{68.4 \pm 14.2}$	64.6 ± 16.6	63.5 ± 20.0	51.8 ± 20.9
mean	64.3 ± 23.0	70.0 ± 15.9	68.8 ± 17.1	$\mathbf{71.2 \pm 18.9}$	65.1 ± 21.4

Table S6. ROC AUC performances in % with standard deviations for *adeno vs. squamous* classification of lung tumor CT scan images *without added contrast*, using radiomic features (*rad*) and topological features (*top*), as well as for three models combining both: through concatenation (*concat*), soft voting (*vote*), and stacking. Each scores is averaged over 50 models, obtained through 10-repeated stratified samplings in 5 folds. Best scores are marked in bold.

Feature correlation for adeno vs. squamous (classification, SF/PA, with contrast)

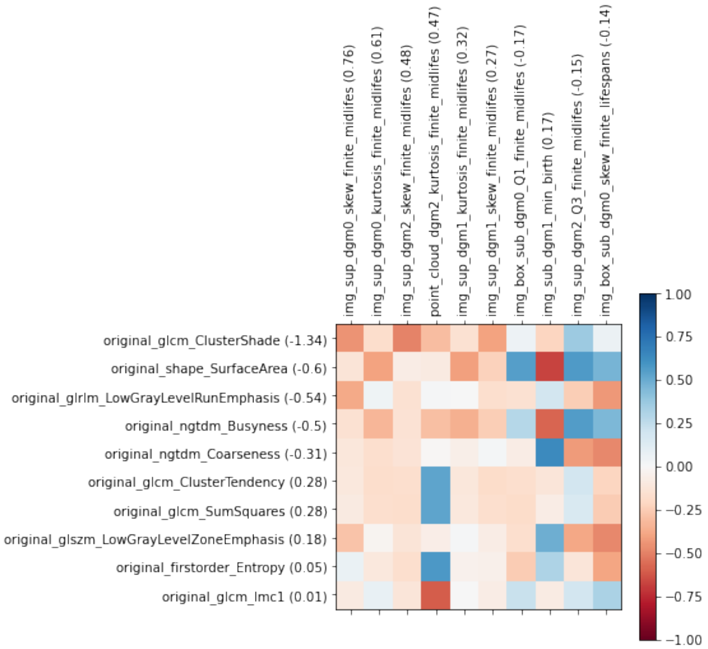

Figure S7. Correlation between the radiomic and topological features selected by the LR models for adeno vs. squamous classification with contrast (SF/PA).

Feature correlation for adeno vs. squamous (classification, SF/PA, without contrast)

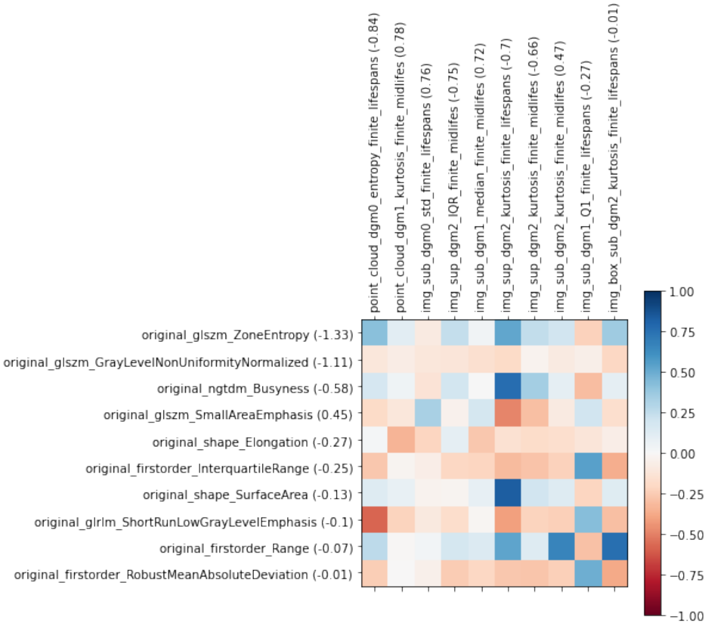

Figure S8. Correlation between the radiomic and topological features selected by the LR models for adeno vs. squamous classification without contrast (SF/PA).

Performances for malignancy prediction (regression, LIDC, with contrast)

model	sem	rad	top	concat	vote	stack
LR	61.3 ± 6.4	57.5 ± 6.1	53.3 ± 5.6	53.3 ± 5.5	58.5 ± 5.5	$\mathbf{58.6 \pm 5.7}$
RF	65.3 ± 6.4	57.3 ± 7.0	54.1 ± 7.0	55.5 ± 6.3	$\mathbf{61.3 \pm 6.1}$	52.6 ± 6.8
KNN	61.6 ± 6.9	57.5 ± 6.4	48.7 ± 10.1	51.8 ± 8.7	$\mathbf{59.4 \pm 6.5}$	51.9 ± 7.3
SV	59.9 ± 7.5	56.4 ± 7.1	51.1 ± 6.0	52.4 ± 5.8	57.5 ± 6.1	$\mathbf{57.6 \pm 6.5}$
BAY	61.4 ± 6.3	57.6 ± 5.9	53.5 ± 5.6	53.6 ± 5.4	58.5 ± 5.5	$\mathbf{58.6 \pm 5.8}$
XGB	57.0 ± 7.3	51.3 ± 9.2	51.4 ± 8.0	53.5 ± 7.1	$\mathbf{59.0 \pm 7.1}$	41.9 ± 8.4
mean	61.1 ± 7.2	56.3 ± 7.4	52.0 ± 7.5	53.4 ± 6.7	$\mathbf{59.0 \pm 6.3}$	53.5 ± 9.0

TABLE S7. r^2 performances in % with standard deviations for continuous *malignancy* outcome prediction of lung tumor nodules from CT scan images *with added contrast*, using semantic features (*sem*), radiomic features (*rad*) and topological features (*top*), as well as for three models combining both: through concatenation (*concat*), soft voting (*vote*), and stacking. Each scores is averaged over 50 models, obtained through 10-repeated samplings in 5 folds. Non-semantic best scores are marked in bold.

Performances for malignancy prediction (regression, LIDC, without contrast)

model	sem	rad	top	concat	vote	stack
LR	54.8 ± 4.9	43.3 ± 5.5	35.6 ± 7.1	36.4 ± 6.9	44.2 ± 5.2	$\mathbf{45.1 \pm 5.3}$
RF	56.9 ± 5.1	45.3 ± 6.0	41.0 ± 8.0	43.6 ± 6.4	$\mathbf{49.0 \pm 5.6}$	36.4 ± 7.9
KNN	54.2 ± 6.1	39.6 ± 7.3	31.8 ± 9.9	35.4 ± 9.4	$\mathbf{45.2 \pm 6.2}$	34.5 ± 8.2
SV	54.1 ± 5.1	42.1 ± 5.8	34.8 ± 7.2	35.5 ± 7.3	43.8 ± 5.4	$\mathbf{44.3 \pm 5.6}$
BAY	54.8 ± 4.9	43.5 ± 5.4	35.7 ± 7.0	36.6 ± 6.9	44.1 ± 5.2	$\mathbf{45.1 \pm 5.3}$
XGB	50.6 ± 5.3	43.0 ± 6.4	39.6 ± 8.1	41.7 ± 6.6	$\mathbf{48.3 \pm 5.7}$	27.4 ± 11.4
mean	54.2 ± 5.6	42.8 ± 6.3	36.4 ± 8.5	38.2 ± 8.0	$\mathbf{45.8 \pm 6.0}$	38.9 ± 10.1

TABLE S8. r^2 performances in % with standard deviations for continuous *malignancy* outcome prediction of lung tumor nodules from CT scan images *without added contrast*, using semantic features (*sem*), radiomic features (*rad*) and topological features (*top*), as well as for three models combining both: through concatenation (*concat*), soft voting (*vote*), and stacking. Each scores is averaged over 50 models, obtained through 10-repeated samplings in 5 folds. Non-semantic best scores are marked in bold.

Feature correlation for malignancy prediction (regression, LIDC, with contrast)

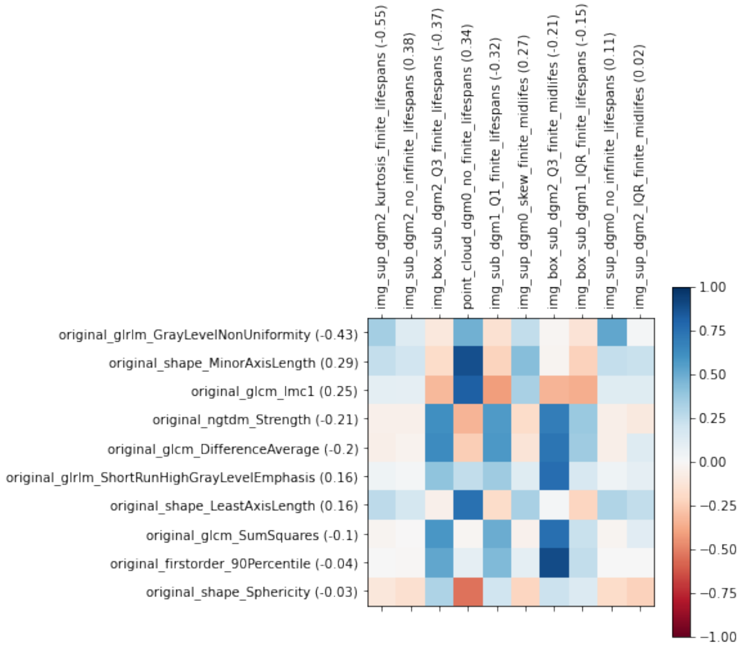

Figure S9. Correlation between the radiomic and topological features selected by the LR models for malignancy prediction with contrast (regression, LIDC).

Feature correlation for malignancy prediction (regression, LIDC, without contrast)

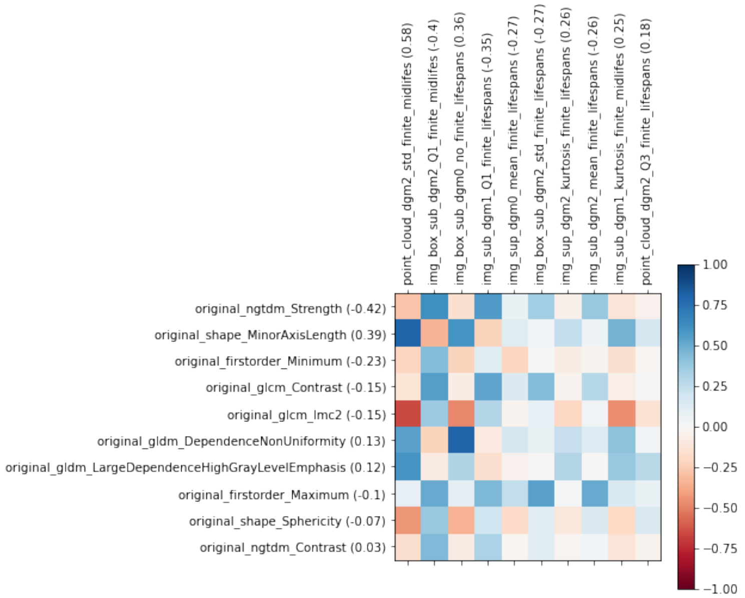

Figure S10. Correlation between the radiomic and topological features selected by the LR models for malignancy prediction without contrast (regression, LIDC).

Performances for benign vs. malignant (classification, LIDC, with contrast)

model	sem	rad	top	concat	vote	stack
LR	66.3 ± 20.2	53.6 ± 19.3	$\mathbf{60.3 \pm 18.9}$	56.4 ± 17.5	57.5 ± 20.2	54.2 ± 20.7
RF	68.2 ± 19.3	57.0 ± 18.4	$\mathbf{61.0 \pm 20.2}$	59.3 ± 19.4	60.6 ± 18.0	50.0 ± 22.1
KNN	66.8 ± 20.1	66.3 ± 17.8	61.7 ± 19.0	62.4 ± 18.6	64.5 ± 18.7	$\mathbf{67.7 \pm 16.4}$
SV	67.7 ± 20.8	56.7 ± 20.6	$\mathbf{59.3 \pm 19.8}$	57.0 ± 15.0	53.9 ± 20.7	52.8 ± 19.7
BAY	64.3 ± 19.5	57.4 ± 20.3	$\mathbf{61.9 \pm 19.0}$	59.7 ± 18.8	61.3 ± 19.4	55.3 ± 22.3
XGB	68.0 ± 16.3	57.9 ± 17.6	$\mathbf{65.7 \pm 17.4}$	61.1 ± 17.8	63.0 ± 17.5	59.5 ± 21.5
mean	66.9 ± 19.5	58.2 ± 19.4	$\mathbf{61.6 \pm 19.2}$	59.3 ± 18.0	60.1 ± 19.5	56.6 ± 21.3

Table S9. ROC AUC performances in % with standard deviations for *benign vs. malignant* classification of lung tumor nodules from CT scan images *with added contrast*, using semantic features (*sem*), radiomic features (*rad*) and topological features (*top*), as well as for three models combining both: through concatenation (*concat*), soft voting (*vote*), and stacking. Each scores is averaged over 50 models, obtained through 10-repeated samplings in 5 folds. Non-semantic best scores are marked in bold.

Performances for benign vs. malignant (classification, LIDC, without contrast)

model	sem	rad	top	concat	vote	stack
LR	16.0 ± 35.3	58.3 ± 46.2	62.0 ± 43.1	$\mathbf{70.3 \pm 42.3}$	53.0 ± 47.7	43.7 ± 43.2
RF	9.3 ± 21.4	57.3 ± 42.6	59.7 ± 41.4	$\mathbf{65.3 \pm 42.7}$	60.0 ± 42.6	24.7 ± 37.0
KNN	29.3 ± 34.6	45.8 ± 33.9	58.0 ± 36.9	$\mathbf{59.5 \pm 37.7}$	52.7 ± 44.7	45.0 ± 32.5
SV	12.0 ± 30.9	$\mathbf{64.3 \pm 45.8}$	56.0 ± 43.2	63.7 ± 43.8	49.0 ± 45.3	51.3 ± 41.3
BAY	14.8 ± 26.2	57.7 ± 40.3	66.5 ± 32.3	64.5 ± 30.5	$\mathbf{76.2 \pm 35.1}$	46.2 ± 43.1
XGB	12.3 ± 21.6	40.8 ± 33.2	76.5 ± 34.8	74.0 ± 38.4	$\mathbf{78.0 \pm 34.9}$	49.0 ± 36.7
mean	15.6 ± 29.6	54.1 ± 41.5	63.1 ± 39.4	$\mathbf{66.2 \pm 39.8}$	61.5 ± 43.5	43.3 ± 40.1

Table S10. ROC AUC performances in % with standard deviations for *benign vs. malignant* classification of lung tumor nodules from CT scan images *without added contrast*, using semantic features (*sem*), radiomic features (*rad*) and topological features (*top*), as well as for three models combining both: through concatenation (*concat*), soft voting (*vote*), and stacking. Each scores is averaged over 50 models, obtained through 10-repeated samplings in 5 folds. Non-semantic best scores are marked in bold.

Feature correlation for benign vs. malignant (classification, LIDC, with contrast)

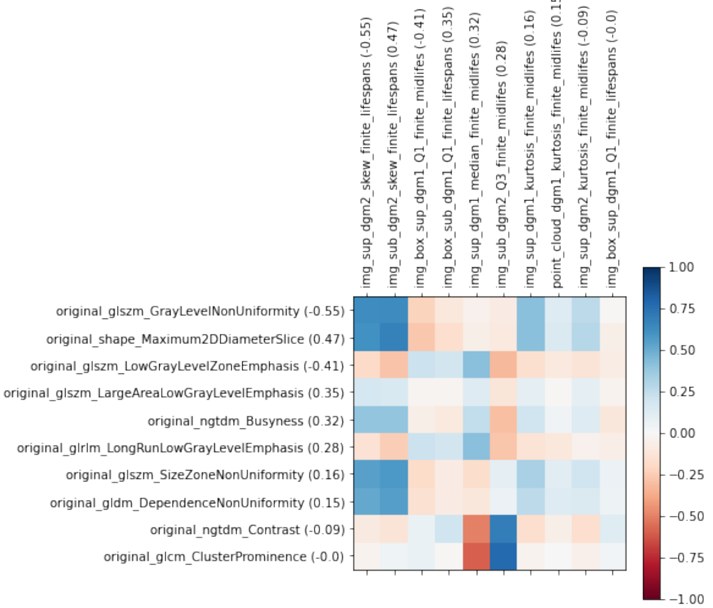

Figure S11. Correlation between the radiomic and topological features selected by the LR models for benign vs. malignant classification with contrast (LIDC).

Feature correlation for benign vs. malignant (classification, LIDC, without contrast)

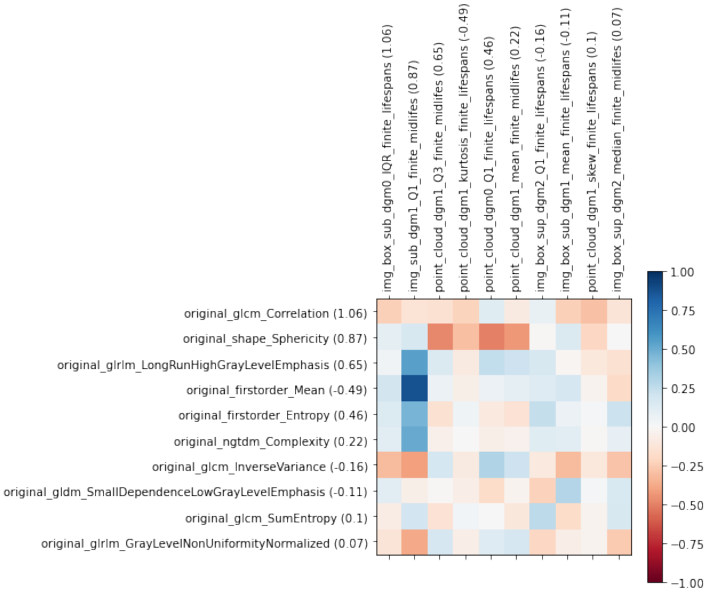

Figure S12. Correlation between the radiomic and topological features selected by the LR models for benign vs. malignant classification without contrast (LIDC).
