# Supplementary material for: Topological data analysis of thoracic radiographic images shows improved radiomics-based lung tumor histology prediction
Source: Patterns (N Y). 2022 Dec 12;4(1):100657. doi: 10.1016/j.patter.2022.100657 (PMC9868648; doi:10.1016/j.patter.2022.100657)
Supplement: Document S2. Article plus supplemental information [file mmc2.pdf]

Article

Patterns

Topological data analysis of thoracic radiographic images shows improved radiomics-based lung tumor histology prediction

Highlights

- We study all of the primary lung tumor classification problems
- We illustrate persistent homology for computed tomography scan images
- We show that topological features may improve radiomic-based histology prediction
- We compare predicting from radiologists' and more accurate histology assessment

Authors

Robin Vandaele, Pritam Mukherjee, Heather Marie Selby, Rajesh Pravin Shah, Olivier Gevaert

Correspondence

robin.vandaele@ugent.be (R.V.), ogevaert@stanford.edu (O.G.)

In brief

We find that topology, a mathematical subject that considers the study of shape, allows one to quantify important information in CT scan images of lung tumors. In this way, tumoral patterns and characteristics that fail to be discovered by the naked eye are discovered. From these, one is able to more rapidly and accurately diagnose the tumor type, which is important for intervention, customized treatment, and monitoring of patients.

Vandaele et al., 2023, Patterns *4*, 100657
January 13, 2023 © 2022 The Author(s).
https://doi.org/10.1016/j.patter.2022.100657

CellPress

Patterns

CellPress
OPEN ACCESS

Article

Topological data analysis of thoracic radiographic images shows improved radiomics-based lung tumor histology prediction

Robin Vandaele,[1,2,3,*] Pritam Mukherjee,[4] Heather Marie Selby,[4,5] Rajesh Pravin Shah,[6,7] and Olivier Gevaert[4,5,8,*]
[1]Department of Applied Mathematics, Computer Science and Statistics, Ghent University, 9000 Ghent, Belgium
[2]Data Mining and Modeling for Biomedicine, VIB Inflammation Research Center, 9052 Ghent, Belgium
[3]IDLab, Department of Electronics and Information Systems, Ghent University, Gent, Belgium
[4]Stanford Center for Biomedical Informatics Research (BMIR), Department of Medicine, Stanford University School of Medicine, Stanford, CA 94305, USA
[5]Department of Biomedical Data Science, Stanford University School of Medicine, Stanford, CA 94305, USA
[6]Veterans Affairs Palo Alto Health Care System, Palo Alto, CA, USA
[7]Department of Radiology, Stanford University, Stanford, CA, USA
[8]Lead contact
*Correspondence: robin.vandaele@ugent.be (R.V.), ogevaert@stanford.edu (O.G.)
https://doi.org/10.1016/j.patter.2022.100657

THE BIGGER PICTURE We study the use of automated features from topological data analysis for all of the most prominent lung tumor histology prediction problems from thoracic images. This is significant because machine learning from computed tomography images requires effective feature engineering methods due to their vast three-dimensional structure and the common small sample sizes in medical cohorts. This is important for rapid diagnosis, intervention, customized treatment, and monitoring of patients. The state of the art considers radiomic features. However, we find that extending these with topological information may improve the accuracy of diagnosis. Finally, while radiomic features appear to be more appropriate for mimicking a radiologist's visual assessment, it turns out that topological features allow more accurate histology prediction, as confirmed through review of radiology images to show 2 years of stable nodule, biopsy, surgical resection, progression, or response.

1 2 3 4 5 **Proof-of-Concept:** Data science output has been formulated, implemented, and tested for one domain/problem

SUMMARY

Topological data analysis provides tools to capture wide-scale structural shape information in data. Its main method, persistent homology, has found successful applications to various machine-learning problems. Despite its recent gain in popularity, much of its potential for medical image analysis remains undiscovered. We explore the prominent learning problems on thoracic radiographic images of lung tumors for which persistent homology improves radiomic-based learning. It turns out that our topological features well capture complementary information important for benign versus malignant and adenocarcinoma versus squamous cell carcinoma tumor prediction while contributing less consistently to small cell versus non-small cell—an interesting result in its own right. Furthermore, while radiomic features are better for predicting malignancy scores assigned by expert radiologists through visual inspection, we find that topological features are better for predicting more accurate histology assessed through long-term radiology review, biopsy, surgical resection, progression, or response.

INTRODUCTION

The recent rise of quantitative imaging in medicine led to new opportunities for assessing severity, change, and disease through quantifiable features from medical images.[1,2] In particular, determining lung cancer histology from computed tomography (CT) scan images is a crucial problem in medical image analysis. Machine-learning models can lead to rapid diagnosis, intervention,

This is an open access article under the CC BY license (http://creativecommons.org/licenses/by/4.0/).

Table 1. The number of observations for each class of lung tumor in the data, with and without added contrast, in the San Francisco/Palo Alto (SF/PA) cohort and the Lung Image Database Consortium (LIDC)

	With contrast	Without contrast	Total
SF/PA			
benign	22	62	84
malignant	33	47	80
small	17	10	27
non-small	16	37	53
adeno	11	20	31
squamous	5	15	20
total	55	109	164
LIDC			
benign	24	5	29
malignant	17	8	25
total	41	13	54

Note that the classes in the SF/PA cohort are not mutually distinct (Figure 1). Here, the last row thus does not equal the sum of the column values.

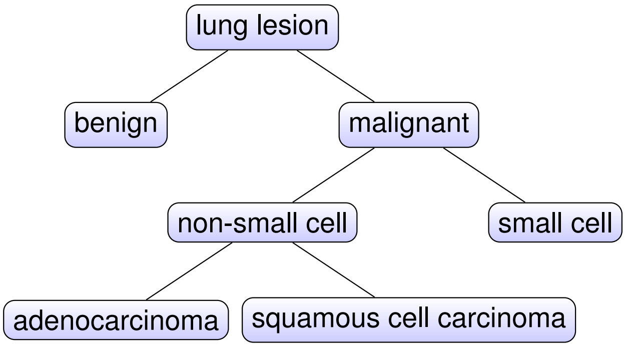

Figure 1. The main hierarchical structure of lung lesions
Each pair of siblings with the same parent in the tree induces a binary classification problem that we study in this article.

customized treatment, and monitoring of patients with lung cancer from such images, reducing the effects of human error in the clinical decision-making process. State-of-the-art models are often based on radiomic features, which cover a wide range of quantitative tumor characteristics such as lesion shape, location, and vascularity.[3]

Complementary to this, the rising field of topological data analysis (TDA)[4] and, in particular, its main method, persistent homology,[5] provide an unparalleled tool to quantify local to global structural information in data. Persistent homology constructs a time-parameterized sequence of combinatorial structures—which can be seen as higher-order generalizations of graphs—from the input data and tracks changes in the topological features—more precisely, holes such as connected components, loops, and voids—along this sequence. These changes are summarized by persistence diagrams, which are sets $\mathcal{D}$ of two-dimensional point coordinates (b,d), marking that a topological feature occurred from birth time b until death time d in the sequence. Persistence diagrams have been effectively incorporated into learning from topological information for various biomedical machine-learning problems. This includes tasks such as predicting protein-protein interaction binding affinity changes,[6] biomedical network classification,[7,8] survival prediction of patients with cancer,[9,10] and skin lesion segmentation.[11] We emphasize that beyond the short introduction to persistent homology given in this paragraph, the many technical and mathematical concepts on which TDA is founded and which are inherent to its introduction are omitted from the main paper. However, a high-level and comprehensive introduction to persistent homology and persistence diagrams that, in particular, focuses on medical image analysis of lung tumors, and which explains how we may consecutively engineer the topological features that are used in this paper, is provided in the supplemental information.

In this article, we study the use of TDA for lung tumor histology prediction from thoracic radiographic images. Our main goal is to study the added value of TDA to all of the prominent learning problems on lung tumor CT scan images compared with state-of-the-art quantitative imaging tools.[12–16]

This retrospective study was approved by the Institutional Review Board overseeing research at both the VA Palo Alto Health Care System and Stanford University. All CT images were obtained from both the Palo Alto and San Francisco VA Picture Archiving and Communication Systems (PACS). We obtained chest CT studies exhibiting cancerous and benign nodules between December 2015 and 2018. For the cancer studies, the inclusion criteria were presence of small cell lung cancer (SCLC), adenocarcinoma (ADC), or squamous cell cancer (SCC). A discussion of the inclusion, exclusion, and size criteria has been previously described.[16] The same criteria were utilized for the San Francisco VA cohort. A CT scan of a primary lung tumor was obtained from each patient, and diagnoses were obtained through biopsy, resection, or serial follow up. Table 1 gives an overview of the number of patients per tumor type. Furthermore, the tumor in each scan was manually delineated by an expert radiologist with greater than 10 years of experience using ITK-SNAP.[17] Figure 2A shows an example of the annotation. Figure 1 displays our three classification problems of interest: each pair of siblings with the same parent in the tree induces a binary classification problem.

Next, we used CT images from 2,544 lung tumor nodules in the Lung Image Database Consortium (LIDC) image collection.[18–20] These nodules include both primary lung tumors as well as metastatic cancers originating from non-lung-tumor sites. The nodules are divided into 807 that were obtained from a scan with contrast material and 1,737 without. The data also contain nodule segmentations provided by multiple expert radiologists for each scan. For tumors with multiple annotations, we used the 50%-consensus segmentation, from which we obtained radiomic and topological features, which will be introduced below. Each annotation included a malignancy score on a discrete scale from 1 to 5 assigned by the expert radiologists. The mean of malignancy scores from the different annotations was considered the "consensus score" for each nodule. Besides the malignancy scores, the radiologists also assigned scores for eight semantic features (SEM): subtlety, internal structure, calcification, sphericity, margin, lobulation, spiculation, and texture.

Table 2. Mean performances in percentage (ROC AUC for classification and r^2 for regression) for lung tumor histology prediction

Problem	C	SEM	Rad	Top	Concat	Vote	Stack	Best model	Best score	p vote ≥ rad
Benign versus	Y	–	84.6	86.8	86.7	87.9[a]	85.8	LR + vote	88.9	$5.7 \cdot 10^{-5}$
malignant (SF/PA)	N	–	74.0	75.7	76.5	78.2[a]	73.8	LR + vote	80.2	$1.7 \cdot 10^{-7}$
Small cell versus	Y	–	77.5[a]	62.7	66.1	75.0	71.7	LR + rad only	79.8	0.94
non-small cell (SF/PA)	N	–	80.6	78.6	80.9	83.4[a]	75.9	RF + vote	86.8	$3.9 \cdot 10^{-2}$
Adeno versus	Y	–	67.2	91.2[a]	90.1	88.3	–	RF + top/concat	98.3	$1.2 \cdot 10^{-17}$
squamous (SF/PA)	N	–	64.3	70.0	68.8	71.2[a]	65.1	BAY + vote	75.0	$3.8 \cdot 10^{-5}$
Malignancy	Y	61.1	56.3	52.0	53.4	59.0[a]	53.5	RF + vote	61.3	$5.6 \cdot 10^{-7}$
regression (LIDC)	N	54.2	42.8	36.4	38.2	45.8[a]	38.8	RF + vote	49.0	$3.3 \cdot 10^{-9}$
Benign versus	Y	66.9	58.2	61.6[a]	59.3	60.1	56.6	KNN + stack	67.7	0.11
malignant (LIDC)	N	15.6	54.1	63.1	66.2[a]	61.5	43.3	XGB + vote	78.0	$1.6 \cdot 10^{-2}$

C, whether contrast material was added (Y) or not (N); SEM, semantic features that were manually assigned by expert radiologists; rad, radiomic features; top, topological features; concat, concatenated radiomic and topological features; vote, voting ensemble; stack, stacking ensemble; p vote ≥ rad, p value for the null hypothesis that the mean performance when using solely radiomic features is at least as good as using both radiomic and topological features through a voting ensemble.
[a]Best mean performances with automated features.

Finally, we considered the histological diagnosis for benign versus malignant tumor classification on a smaller LIDC data sample of 54 primary lung tumor nodules for which true diagnoses were available. These were obtained based on one of the following criteria: review of radiology images to show 2 years of stable nodule, biopsy, surgical resection, progression, or response.[18–20] Table 1 summarizes the number of patients per primary tumor type, with and without added contrast material, for which diagnoses were available.

The main contributions of this work are as follows. Through a cohort of patients with lung cancer from multiple institutes including Stanford and several VA hospitals, we compare topological-features-based classifications with standard radiomic-features-based classifications of lung tumors with and without contrast material. In particular, we study all of the main lung tumor classification problems that can be considered: "benign versus malignant," "small cell versus non-small cell," and "ADC versus squamous cell carcinoma" (Figure 1). We show that topological features consistently provide additional and valuable information when combined with radiomic features for benign versus malignant classification and adeno versus squamous while, interestingly, contributing less consistently to small cell versus non-small cell. Furthermore, the enhanced performance for malignancy prediction is confirmed for both a binary and a continuous outcome on the LIDC image collection.[18–20] Finally, we discuss further directions for studying and improving lung tumor histology prediction through TDA.

RESULTS

All results for the various histology prediction problems are summarized in Table 2. Rows correspond to the considered classification or regression problem. More detailed tables can be found in the supplemental information (Tables S1–S10). We also include correlation matrices as well as feature importances for the features selected by the pipelines including a logistic/linear regression (LR) model (Figures S3–S12), which were consistently among the better-performing models, either on their own or through a soft-voting ensemble.

Lung tumor histology prediction (San Francisco/Palo Alto [SF/PA])

We considered three binary classification problems to evaluate TDA and compare it with radiomics-based lung tumor histology prediction: benign versus malignant, small cell versus non-small cell, and squamous versus ADC. For benign versus malignant, we see that adding topological features generally improves solely radiomic-features-based classification. In particular, using solely topological features already often outperforms radiomic features for classification. Nevertheless, the voting ensemble using both types of features consistently leads to the best performances. Topological features have the strongest (either strongly positive or strongly negative) correlation with radiomic features for scans with contrast, whereas they are less correlated for scans without contrast (Figures S3 and S4).

Next, for the small cell versus non-small cell classification problem, we observe that the topological features do not perform as well as radiomic features both for scans with and without added contrast. However, for images without added contrast, topological features do contribute to the final prediction model through a voting ensemble. Interestingly, both types of features appear to favor scans without contrast material for this particular classification problem. However, the performance differences between the base models with and without contrast are significantly higher for topological features than for radiomic features. It is noteworthy that there is little correlation between topological and radiomic features for scans with contrast, whereas some correlation can be found for scans without contrast (Figures S5 and S6). This may explain why the models using the different types of features are more on par for scans without contrast (Table 2).

Finally, for the ADC versus squamous cell carcinoma classification problem, we observe the highest performance increases when using topological features, most significantly for images with added contrast. Topological features perform both much better on their own as well as combined with radiomic features through concatenation or a voting ensemble. Interestingly, there is significantly more performance difference between with and without contrast material for topological features than for

radiomic features. With a few exceptions, there is little correlation between the selected radiomic and topological features (Figures S7 and S8).

Lung tumor malignancy prediction from radiologists' assessment (LIDC)

The outcome here corresponds the continuous malignancy scores assigned by the radiologist for the 2,544 lung tumor nodules in the LIDC image collection, 807 scan with contrast material, and 1,737 without. Recall that these were made by the radiologist based on their visual assessments of a set of eight semantic features. For this outcome, the semantic features (which we averaged over different expert annotations) can thus, in some sense, be considered optimal for prediction. Naturally, there still remains variance in the outcome that is unexplained by solely the semantic features, e.g., due to averaging over the feature and outcome assessments made by different radiologists.

Considering the performances of the automated features, we observe that both with and without contrast material, radiomic features overall perform better than topological features. Thus, the radiomic features appear more applicable to mimic the manual predictions made by the radiologists. Nevertheless, we observe consistent improvements when combining radiomic with topological features through a voting ensemble.

Lung tumor histology prediction from pathology ground truth (LIDC)

Finally, as discussed above, the manual predictions made by the radiologist are not always representative for the true lung tumor histology. We observe that while radiomic features were better at predicting the radiologist's manual outcome annotations, topological features are actually better at predicting the accurate tumor diagnoses—at least on this smaller portion of the data for which they are available. Another interesting observation is that while the manually annotated semantic features perform best for images with contrast, they reach poor performance on images without contrast—unlike topological features. A possible explanation is that while the semantic features are valuable for predicting histology, their visual assessment may be more difficult without contrast.

DISCUSSION

In this article, we studied the use of topological features for various lung tumor histology classification and regression problems. In particular, we included a thorough overview for all principal prediction problems that might be considered from thoracic radiographic images of the lungs, further splitting our analysis into scans with and without added contrast material.

Our results consistently suggest that TDA provides a promising approach to lung tumor histology prediction from thoracic radiographic images, most notably for benign versus malignant and adeno versus squamous classification. Furthermore, as discussed in the supplemental information, we use a straightforward vectorization through summary statistics to obtain our topological features. Unavoidably, one loses information when transforming topological information into feature representations suitable for machine learning through such a (or any other) process. Therefore, a variety of complementary ways to learn through TDA exists. These already found successful machine-learning applications in medicine, in particular oncology, for example, to predict the survival prognosis of patients with lung cancer,[9,21] predict the disease-free survival of patients with glioblastoma multiforme brain cancer,[10] or analyze the heterogeneity in 3D thoracic CT images.[22] Note that we do not claim our used topological feature extraction method to be superior to the methods described in these papers as we suggest investigating their applicability to the parallel problems that we studied in this article as further research. Thus—even though we already achieved encouraging results in this article—given the extensive manners to learn from TDA, much of its true potential for rapid diagnosis, intervention, customized treatment, and monitoring of patients with lung cancer is yet to be uncovered.

Furthermore, the contributions of our work are notably different from those in other recent studies on TDA for lung tumor histology prediction. For example,[21] it focuses on survival analysis of non-SCLC while[22] applying TDA to summarize textural information of lung ADCs. In contrast to this, our goal was to objectively study the potential of topological analysis for all primary lung tumor type classification problems, a study that, to the best of our knowledge, has not yet been performed. The fact that earlier work also demonstrates the effectiveness of TDA for (lung) tumor analysis further supports our findings on the Stanford unique data.

Beyond our main focus on the added effect of TDA for lung tumor histology prediction, our thorough performance evaluations led to extensive quantitative summarizations that are genuinely valuable. These include deeply interesting results open to further exploration, such as the different effects of contrast material or the performance differences between semantic, radiomic, and topological features when predicting the malignancy scores assigned by the radiologist versus when predicting the true tumor histology. For example, we found that while radiomic features may be more suitable for mimicking the radiologists' malignancy annotations, topological features may actually be more suitable for predicting the true tumor histology.

EXPERIMENTAL PROCEDURES

Resource availability

Lead contact

Further information and requests for resources should be directed to the lead contact, Olivier Gevaert (ogevaert@stanford.edu).

Materials availability

No new materials were generated by this study.

Data and code availability

Data to replicate the results summarized in this paper are available from GitHub:robinvndaele.[23] This includes persistence diagrams, features, metadata, and outcomes for both the SF/PA and LIDC data. Original scans and masks for the SF/PA cohort are excluded from this repository and are not permitted to be shared. Original LIDC scans and masks are publicly available from TCIA: https://doi.org/10.7937/K9/TCIA.2015.LO9QL9SX. All code for this project is available on GitHub:robinvndaele.[23]

Quantitative image features extraction

Radiomic features

All images and masks were resampled to 1 × 1 × 1 mm^3. Radiomic features were then extracted using PyRadiomics 1.0[24] from the defined regions of interest. We selected 105 Image Biomarker Standardization Initiative (IBSI) 11-compliant features across the following classes: first order statistics

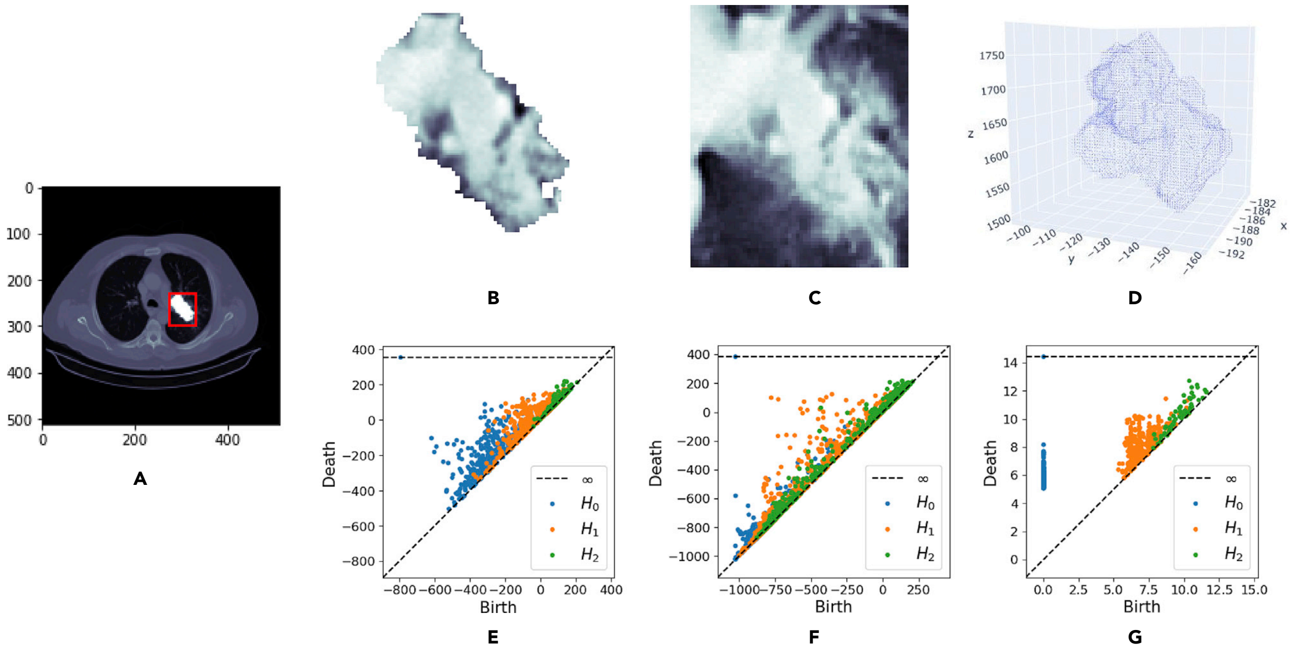

Figure 2. Different ways to compute persistent homology from thoracic radiographic images

(A) An example 2D slice of a lung tumor CT scan. Pixel values—which can range on a grayscale from black to white—capture the radiodensity of the material at the location. Brighter pixels correspond to radiodense material that more inhibits the passage of radiation (X-rays), such as bones or tumor tissue. Darker pixels correspond to radiopaque material that allows radiation to pass more freely, such as muscle and skin. The tumor in the lungs is marked by a red boundary box. The segmented tumor pixels are highlighted in white, not to be confused with their CT pixel values or, thus, radiodensity values, which are better visualized in the following two images.

(B) The same slice of the CT scan image but restricted to the segmented tumor pixels.

(C) The same slice of the CT scan image but restricted to the pixels in the boundary box of the segmented tumor.

(D) A point cloud representing the tumor surface in $\mathbb{R}^3$. It can be defined by stacking the tumor contours of all 2D slices of the CT scan.

(E) The persistence diagrams obtained from the sublevel filtration of the 3D tumor image, of which a 2D slice is shown on image (B) above. Three persistence diagrams are plotted on top of each other. There is one diagram for each of the three dimensions of topological hole considered (H0/0-dim.: components, H1/1-dim.: cycles, H2/2-dim.: voids).

(F) The persistence diagrams obtained from the sublevel filtration of the 3D tumor image with surrounding boundary box pixels, of which a 2D slice is shown in (C) above.

(G) The approximated persistence diagrams for the Vietoris-Rips filtration of the point cloud representing the tumor surface in $\mathbb{R}^3$, shown in (D) above.

(19 features), shape-based (3D) (16 features), gray-level size zone matrix (GLSZM) (16 features), gray-level co-occurrence matrix (GLCM) (24 features), gray-level run length matrix (GLRLM) (16 features), gray-level size zone matrix (GLSZM) (16 features), neighboring gray tone difference matrix (NGTDM) (5 features), and gray-level dependence matrix (14 features).

Topological features

From each scan, we obtained different types of persistence diagrams. These diagrams, discussed in detail in the supplemental information, quantify topological holes in combinatorial objects termed simplicial complexes, which are higher-order generalizations of graphs that are constructed from the scan that grows (thus includes more simplices) with some time parameter t. This quantification is performed through birth-death pairs (b,d), which characterize a topological hole that appeared (was born) at time $t = b \in \mathbb{R}$ and that (possibly never) disappeared (died), a time $t = d \in \mathbb{R} \cup \{\infty\}$. Topological holes in the lesion, and thus persistence diagrams, can be distinguished by their dimension: connected components (dimension 0), cycles (dimension 1), and voids (dimension 2). These persistence diagrams can furthermore be distinguished by the object through which they capture topological information from the tumor. We considered five such objects, namely the lesion pixels (raw and negated), the lesion pixels with boundary box pixels (raw and negated), and a point cloud representing the lesion surface. Hence, from each scan, we obtained 5 × 3 = 15 persistence diagrams: 3 dimensions of holes for each of the 5 objects. Figure 2 illustrates 3 × 3 examples of such diagrams and the objects they are computed from. Finally, we obtained summary statistics from these diagrams, as detailed below, resulting in a vector of 290 topological features per scan.

Supervised machine-learning modeling

For each of our machine-learning models (discussed hereafter), we used the same feature preprocessing, consisting of the following steps: (1) missing values (which rarely occurred when a persistence diagram was empty) were imputed with the mean; (2) features were minimum-maximum (min-max) scaled to [0, 1]; (3) features were binned into five partitions of equal length, which serves the following feature selection method that takes discretized features; and (4) to maintain a similar number of radiomic and topological features and to reduce the effects of overfitting, we used a feature selection procedure based on minimum redundancy maximum relevance (mRMR) to select 10 features for the final prediction model.[25] We then combined this preprocessing pipeline with each one of six commonly applied classification or regression models (depending on the outcome), namely logistic/linear regression (LR), random forest classification/regression (RF), k -nearest neighbor classification/regression (KNN), support vector machine/regressor (SV), Gaussian naive Bayes classification/Bayesian regression (BAY), and extreme gradient-boosted trees classification/regression (XGB). Note that the same abbreviation may represent a different machine-learning model depending on whether classification or regression is considered and that most abbreviations are only

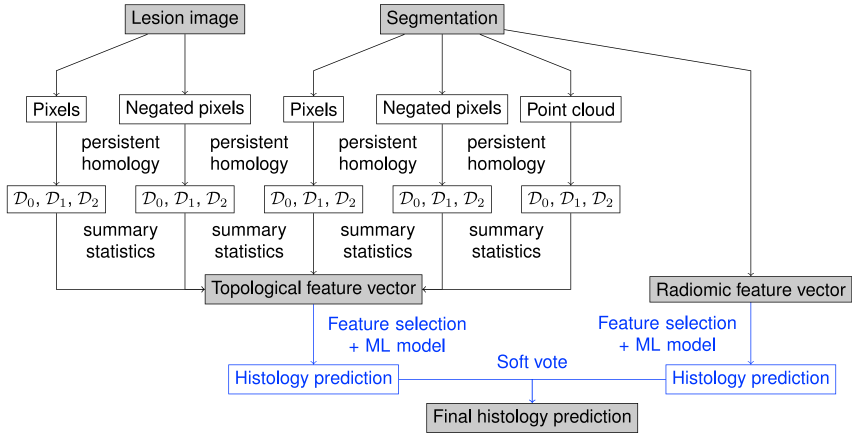

Figure 3. Schematic overview of the machine-learning pipeline we present in this article

The part shown in blue may vary depending on which type of features we use or how they are combined. The example shown is for a voting ensemble that uses both types of features.

used in Tables S1–S10. Finally, to compare radiomic and topological features, we used various types of feature and model combinations to evaluate the histology prediction models, namely training on radiomic features only (rad); training on topological features only (top); training on the concatenated features (concat); a soft-voting ensemble using models trained on the radiomic and topological features separately (vote); and a stacking ensemble, which is—unlike the voting ensemble that combines the output directly—retrained on the probabilistic output of the models that where trained on both feature types separately (stack). The stacking estimator was equal to the base estimators, e.g., if the base estimators were logistic regression models then so was the final estimator. Note that there were insufficient scans with contrast of squamous tumors in the SF/PA cohort to evaluate a stacking classifier through cross-validation.

Figure 3 shows a schematic overview of the machine-learning pipeline we applied to evaluate and compare topological and radiomic features for lung tumor histology prediction.

Model evaluation

To evaluate the models and thus the features, we used 10 repeats of 5-fold cross-validation to measure the performance of the classification (receiver operating characteristic [ROC] area under the curve [AUC]) and regression (r^2 coefficient of determination) models. Per classification/regression problem, the folds were kept the same over all model evaluations. For the classification problems on the SF/PA data, we used stratified sampling to obtain the folds. This is especially important given the small sample sizes, e.g., to ensure that each fold contains examples of both classes. For the regression problems on the LIDC data, we used standard random sampling. However, this sampling was conducted on the patient level rather than the nodule level, ensuring that different nodules from the same patient were within the same fold. For the classification problems on the LIDC data, we also used stratified sampling. For this, we constructed a class variable indicating whether a patient had a benign tumor nodule (only rarely did a patient have both benign and malignant nodules) as to again be able to perform the sampling on the patient level. Note that this class variable was not used for the final outcome as the lung tumor histology predictions were made on the nodule level.

SUPPLEMENTAL INFORMATION

Supplemental information can be found online at https://doi.org/10.1016/j.patter.2022.100657.

ACKNOWLEDGMENTS

The research leading to these results has received funding from the FWO (project no. V407520N, G091017N, G0F9816N, and 3G042220), the European Research Council under the European Union's Seventh Framework Programme (FP7/2007–2013)/ERC Grant agreement no. 615517, and the Flemish government under the "Onderzoeksprogramma Artificiële Intelligentie (AI) Vlaanderen" program. Next, research reported in this publication was supported by the National Institute of Biomedical Imaging and Bioengineering (NIBIB) of the National Institutes of Health (NIH), R01 EB020527 and R56 EB020527, both to O.G. This material is the result of work supported with resources and the use of facilities at the VA Palo Alto Health Care System (Palo Alto, CA, USA). The content is solely the responsibility of the authors and does not necessarily represent the official views of the NIH.

AUTHOR CONTRIBUTIONS

R.V. performed the machine-learning experiments and TDA and wrote the main manuscript. P.M. and H.M.S. provided radiomic features, were included in the discussion of the results, and edited the manuscript. R.P.S. performed data collection and study design. O.G. supervised the entire project.

DECLARATION OF INTERESTS

The authors declare no competing interests.

Received: May 14, 2022
Revised: July 15, 2022
Accepted: November 15, 2022
Published: December 12, 2022

REFERENCES

1. Gatenby, R.A., Grove, O., and Gillies, R.J. (2013). Quantitative imaging in cancer evolution and ecology. Radiology *269*, 8–15.
2. Acharya, U.R., Hagiwara, Y., Sudarshan, V.K., Chan, W.Y., and Ng, K.H. (2018). Towards precision medicine: from quantitative imaging to radiomics. J. Zhejiang Univ. - Sci. B *19*, 6–24.
3. Ranjbar, S., and Mitchell, J.R. (2017). An introduction to radiomics: an evolving cornerstone of precision medicine. In Biomedical Texture Analysis, A. Depeursinge, O.S. Al-Kadi, and J.R. Mitchell, eds. (Elsevier), pp. 223–245.
4. Carlsson, G. (2009). Topology and data. Bull. Amer. Math. Soc. *46*, 255–308.
5. Ghrist, R. (2007). Barcodes: the persistent topology of data. Bull. Amer. Math. Soc. *45*, 61–76.
6. Wang, M., Cang, Z., and Wei, G.-W. (2020). A topology-based network tree for the prediction of protein–protein binding affinity changes following mutation. Nat. Mach. Intell. *2*, 116–123.
7. Carrière, M., Chazal, F., Ike, Y., Lacombe, T., Royer, M., and Umeda, Y. (2020). Perslay: a neural network layer for persistence diagrams and new graph topological signatures. In International Conference on Artificial Intelligence and Statistics (PMLR), pp. 2786–2796.
8. Hofer, C., Graf, F., Rieck, B., Niethammer, M., and Kwitt, R. (2020). Graph filtration learning. In International Conference on Machine Learning (PMLR), pp. 4314–4323.

CellPress
OPEN ACCESS

9. Kadoya, N., Tanaka, S., Kajikawa, T., Tanabe, S., Abe, K., Nakajima, Y., Yamamoto, T., Takahashi, N., Takeda, K., Dobashi, S., et al. (2020). Homology-based radiomic features for prediction of the prognosis of lung cancer based on ct-based radiomics. Med. Phys. *47*, 2197–2205.
10. Crawford, L., Monod, A., Chen, A.X., Mukherjee, S., and Rabadán, R. (2020). Predicting clinical outcomes in glioblastoma: an application of topological and functional data analysis. J. Am. Stat. Assoc. *115*, 1139–1150.
11. Vandaele, R., Nervo, G.A., and Gevaert, O. (2020). Topological image modification for object detection and topological image processing of skin lesions. Sci. Rep. *10*, 21061.
12. Coroller, T.P., Grossmann, P., Hou, Y., Rios Velazquez, E., Leijenaar, R.T.H., Hermann, G., Lambin, P., Haibe-Kains, B., Mak, R.H., and Aerts, H.J.W.L. (2015). Ct-based radiomic signature predicts distant metastasis in lung adenocarcinoma. Radiother. Oncol. *114*, 345–350.
13. Coroller, T.P., Agrawal, V., Narayan, V., Hou, Y., Grossmann, P., Lee, S.W., Mak, R.H., and Aerts, H.J.W.L. (2016). Radiomic phenotype features predict pathological response in non-small cell lung cancer. Radiother. Oncol. *119*, 480–486.
14. Bakr, S., Gevaert, O., Echegaray, S., Ayers, K., Zhou, M., Shafiq, M., Zheng, H., Benson, J.A., Zhang, W., Leung, A.N.C., et al. (2018). A radiogenomic dataset of non-small cell lung cancer. Sci. Data *5*, 180202–180209.
15. Brunese, L., Mercaldo, F., Reginelli, A., and Santone, A. (2019). Neural networks for lung cancer detection through radiomic features. In 2019 International Joint Conference on Neural Networks (IJCNN) (IEEE), pp. 1–10.
16. Shah, R.P., Selby, H.M., Mukherjee, P., Verma, S., Xie, P., Xu, Q., Das, M., Malik, S., Gevaert, O., and Napel, S. (2021). Machine learning radiomics model for early identification of small-cell lung cancer on computed tomography scans. JCO Clin. Cancer Inform. *5*, 746–757.
17. Yushkevich, P.A., Piven, J., Hazlett, H.C., Smith, R.G., Ho, S., Gee, J.C., and Gerig, G. (2006). User-guided 3d active contour segmentation of anatomical structures: significantly improved efficiency and reliability. Neuroimage *31*, 1116–1128.
18. Clark, K., Vendt, B., Smith, K., Freymann, J., Kirby, J., Koppel, P., Moore, S., Phillips, S., Maffitt, D., Pringle, M., et al. (2013). The cancer imaging archive (tcia): maintaining and operating a public information repository. J. Digit. Imaging *26*, 1045–1057.
19. Armato, S.G., III, McLennan, G., Bidaut, L., McNitt-Gray, M.F., Meyer, C.R., Reeves, A.P., Zhao, B., Aberle, D.R., Henschke, C.I., Hoffman, E.A., et al. (2011). The lung image database consortium (lidc) and image database resource initiative (idri): a completed reference database of lung nodules on ct scans. Med. Phys. *38*, 915–931.
20. Armato, III, Samuel, G., McLennan, G., Bidaut, L., McNitt-Gray, M.F., Meyer, C.R., Reeves, A.P., Zhao, B., Aberle, D.R., Henschke, C.I., et al. (2015). Data from Lidc-Idri. https://wiki.cancerimagingarchive.net/x/rgAe.
21. Somasundaram, E., Litzler, A., Wadhwa, R., Owen, S., and Scott, J. (2021). Persistent homology of tumor ct scans is associated with survival in lung cancer. Med. Phys. *48*, 7043–7051.
22. Kawata, Y., Niki, N., Kusumoto, M., Ohamatsu, H., Aokage, K., Ishii, G., et al. (2021). Representation of texture structures with topological data analysis for stage ia lung adenocarcinoma in three-dimensional thoracic ct images. In Medical Imaging 2021: Biomedical Applications in Molecular, Structural, and Functional Imaging, *11600*, B.S. Gimi and A. Krol, eds. (SPIE), pp. 106–112.
23. robinvndaele (2022). robinvndaele/TDA_LungLesion: First Release for Publication. https://doi.org/10.5281/zenodo.7294728.
24. van Griethuysen, J.J.M., Fedorov, A., Parmar, C., Hosny, A., Aucoin, N., Narayan, V., Beets-Tan, R.G.H., Fillion-Robin, J.-C., Pieper, S., and Aerts, H.J.W.L. (2017). Computational radiomics system to decode the radiographic phenotype. Cancer Res. *77*, e104–e107.
25. Ding, C., and Peng, H. (2005). Minimum redundancy feature selection from microarray gene expression data. J. Bioinform. Comput. Biol. *3*, 185–205.

Patterns, Volume *4*

Supplemental information

Topological data analysis of thoracic radiographic images shows improved radiomics-based lung tumor histology prediction

Robin Vandaele, Pritam Mukherjee, Heather Marie Selby, Rajesh Pravin Shah, and Olivier Gevaert

SUPPLEMENTAL INFORMATION

Supplemental experimental procedures

Persistent homology. *Persistent homology* is unarguably the most studied and applied method in topological data analysis (TDA). Its roots are in the field of *algebraic topology*[S1], where it has been developed to quantify changes in topological *holes* across a *filtration*, i.e., an ordered sequence of *simplicial complexes*

$$\mathcal{F} = K_0 \subseteq K_1 \subseteq \ldots \subseteq K_N = K$$

of an initial complex K. A simplicial complex K can be seen as a generalization of a graph, that apart from nodes (0-simplices) and edges (1-simplices), also includes *higher-dimensional simplices* such as triangles (2-simplices), tetrahedra (3-simplices), $\ldots$, with the constraint that if K contains a simplex σ, every simplex $\sigma' \subseteq \sigma$ must also be contained in K. Figure S1a illustrates an example of such a filtration.

The topological holes that are quantified through persistent homology, are characterized by their dimension as follows.

- 0-dimensional holes correspond to gaps between connected components.
- 1-dimensional holes correspond to the inside of a loop, such as the inside of a ring or the handle of a coffee mug.
- 2-dimensional holes correspond to voids, such as the inside of a balloon.
- In general, a k-dimensional hole corresponds to the inside of a k-sphere. They can only occur in a space of at least dimension $k+1$. For $k \geq 3$, these holes become difficult to visualize. These holes are also not used in this paper, since they never occur in the mathematical objects from which we compute persistent homology.

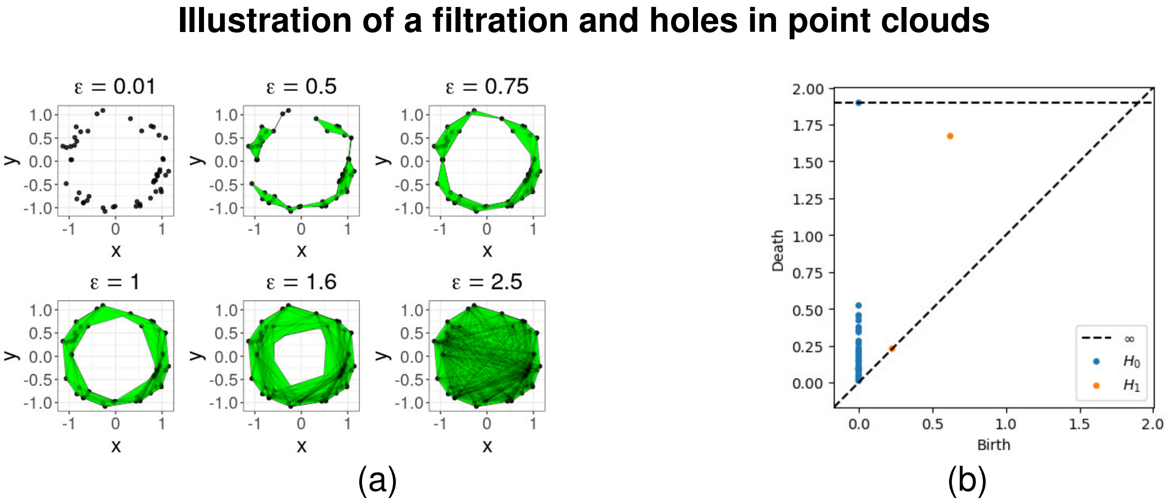

FIGURE S1. (A) An example of various simplicial complexes in a filtration constructed from a point cloud dataset. Here, the filtration equals the Vietoris-Rips filtration, parameterized by a time (distance) parameter ϵ. At time ϵ, all simplices with at most three nodes and a diameter—that is, the maximal pairwise distance between the nodes of the simplex—of at most ϵ are included in the complex. (B) The two corresponding persistence diagrams, one for each considered dimension of hole, plotted on top of each other. Note that this example does not relate to our tumor imaging data: it is mainly used as an interpretable illustration of what persistent homology is able to quantify. The two most elevated points quantify the connected component (H0) and cycle (H1) in the underlying circular model of the data, as explained below. Observe that for our application however, we also aim to learn from topological information on a smaller scale, not only from the most prominent holes.

The number of k-dimensional holes of a simplicial complex is denoted by the *Betti-number* β_k. In particular, β_0 denotes the number of connected components.

Given a simplicial complex K, and a real-valued function f defined on all simplices in K, a filtration can generally be written in the form of a *sublevel filtration*

$$\mathcal{F} = \{\sigma \in K : f(\sigma) \leq t\}_{t\in\mathbb{R}},$$

parameterized by a *time* parameter t. Note that the term 'time' is to be loosely interpreted, as the parameter can also capture another unit such as distance (Figure S1) or pixel intensity (Figure S2). In practice, i.e., when dealing with finite data, simplicial complexes in $\mathcal{F}$ change for only finitely many values $t_0, \ldots, t_N \in \mathbb{R}$. E.g., the filtration in Figure S1a equals the *Vietoris-Rips* filtration, where K contains all subsets of a given metric space, i.e., a point cloud dataset, and f maps each subset to its diameter. In this case the dataset is 3-dimensional, and hence, no holes of dimension 3 or higher can occur. Thus, we do not include simplices that are higher-dimensional, i.e., include more points, than triangles. The Vietoris-Rips filtration is used to obtain topological information from the point cloud modeling the tumors surfaces (Figures 2d and 2g).

A filtration can also be obtained directly from the CT scan image pixels of a tumor, quantifying the radiodensity of the tissue. Note that such scan can be regarded as a 3D array of pixels, of which an example slice restricted to the tumor pixels is shown in Figure 2b. By connecting neighboring pixels, that is, pixels that are horizontally, vertically, or diagonally adjacent to each other, in a particular manner, we obtain a three-dimensional complex K that is known as *Fruedenthal's triangulation*[1S4]. The original image can now be regarded as a real-valued function f defined on the 0-simplices in K, where for a 0-simplex (pixel) p, $f(p)$ denotes the intensity of p in the original image. Through this function, we can define a sublevel filtration directly from the pixel values of the original image as

$$\mathcal{F} = \left\{\sigma \in K : f(\sigma) \coloneqq \max_{p\in\sigma}(p) \leq t\right\}_{t\in\mathbb{R}}.$$

Intuitively, the complex at time t is induced by all pixels with intensity at most t, and their neighboring relations. The resulting filtration for the image in Figure 2b is shown in Figure S2a. By being inherently 3-dimensional, only up to 2-dimensional holes (voids) can occur in the filtration. The range of our filtration is determined by the minimum and maximum pixel value, and thus is chosen in a parameter-free manner.

For both types of filtrations, whether derived from a point cloud or image pixels, persistent homology tracks the *birth* (b) and *death* (d) of these holes across the filtration. The obtained tuples (b, d) are then commonly visualized by means of a *persistence diagram*, one for each dimension of hole. E.g., Figure S1b shows the persistence diagrams of the Vietoris-Rips filtration in Figure S1a. In this filtration, every point defines the birth of a connected component at time $\epsilon = 0$. These correspond to the blue points in Figure S1b (H0). By connecting more and more distant points by edges, and 'filling in' the resulting triangles, we see that many connected components die (they merge with others). Eventually a 1-dimensional hole (a circle) is formed by the complex, which persists for a relatively long time in the filtration, and finally gets filled in and thus dies. This circle is marked by the highly elevated orange point (H1) in Figure S1b. For the filtration in Figure S2a (of which the persistence diagrams are shown in Figure 2e), the darkest pixels correspond to connected components that are born first. When brighter pixels are consecutively added during the filtration, they may either give rise to new connected components (which are then born as well), or immediately connect to darker pixels which were already present. In the latter case, they may also merge two previously disconnected components, resulting in the death of a connected component. Although 1- and 2-dimensional holes may also be born and die (or even persist indefinitely) during this filtration, this is less apparent from Figure S2a. This becomes more intuitive from Figure S2b.

[1]Another popular type of complex for image data is the *cubical complex*[S2]. It can be straightforwardly defined on a 3D image by connecting all neighboring pixels that are either horizontally or vertically adjacent to each other. The connected points then form the edges, squares, and cubes of which the complex is composed. Cubical complexes are thus similar to simplicial complexes, but with hypercubes instead of hypertriangles. Using cubical complexes to compute persistent homology from images is standard practice[S3]. We opted for the Fruedenthal's triangulation however because of our familiarity with the Dionysus library in Python in which it was readily available. By no means however do we claim Fruedenthal's triangulation to be superior over cubical complexes for this application. We do not expect significant differences when applying the different types of complexes for our application, as they are meant to capture similar topological information.

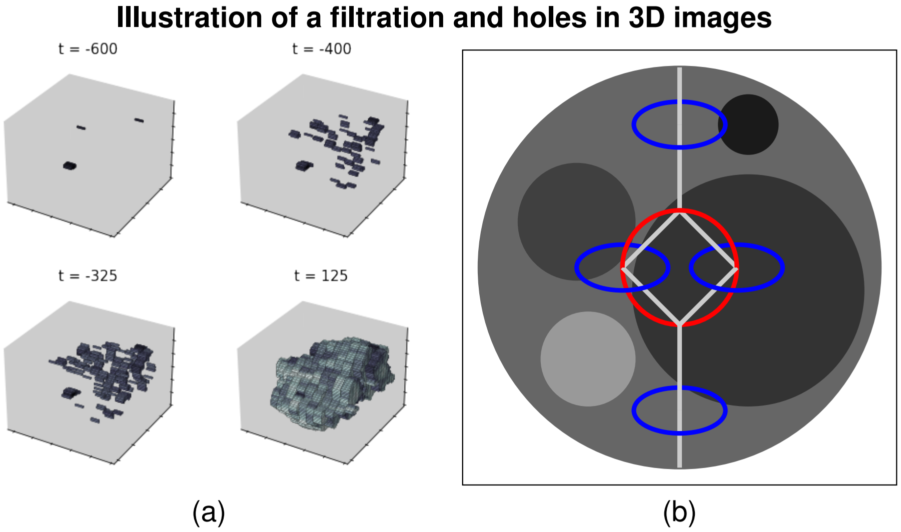

Figure S2. (A) An example of various simplicial complexes in a filtration constructed from the segmented 3D tumor image of which one 2D slice is shown in Figure 2a. Here, the filtration equals the sublevel filtration defined by the function f mapping each pixel (0-simplex) to its value in the original CT scan image. Darkest pixels are included first. By increasing the time parameter t, increasingly brighter pixels are included in the complex as well. 1-, 2-, and 3-simplices are induced by the neighboring relationships between pixels, and not shown in this illustration. (B) A simplified artificial illustration of how various holes may occur in a 3D grayscale image. The illustration should be imagined as representing the restrictions of the holes to a 2D-slice of a 3D-image. The largest sphere represents a lesion, the smaller enclosed spheres tissue components of varying radiodensity within the lesion, and the white lines blood vessels that would appear brighter on scans with contrast material. In a sublevel filtration, the darkest pixels are included first. The enclosed spheres that are darker than the surrounding pixels will thus be included first as separate components, until they merge when the brighter surrounding pixels are included as well. These components will thus be detected in the 0-dimensional persistence diagram of the sublevel filtration, where their birth time b represents the radiodensity of the tissue component, and their death time d represents the radiodensity of the surrounding tissue. Note that these components would similarly be detected as voids when brighter pixels would have been included first, i.e., through the 2-dimensional persistence diagram of the superlevel filtration. An analogous explanation holds for enclosed spheres that are brighter than the surrounding pixels. In case of 1-dimensional persistence, the holes surrounding the blood vessels (blue circles) will be discovered through the sublevel filtration, as the vessels themselves will be excluded from the filtration until the brightest pixels are included at the end of the filtration. The holes defined by the vessels themselves (red circle) will be discovered through the superlevel filtration, as the interior tissue would be excluded until the darker pixels are included. Unlike radiomic features which may also capture properties such as tissue homogeneity and vascularity, persistent homology quantifies topological properties over the entire (color) scale of the data. Our topological features summarize their distribution across this scale.

Learning from persistence diagrams. One of the main original ideas behind persistent homology and persistence is that holes persisting for a long time–which correspond to 'highly elevated' points in their respective persistence diagram—-represent significant features of the underlying topology (hence the name 'persistence'). E.g., this in Figure S2a the single highly elevated point for connected components (H0) represents that the underlying topological model is connected, and the single highly elevated point for loops (H1) that the underlying topological model contains a cycle.

More recently it has been shown that the entire distribution of points on a persistence diagram may play a significant role in characterizing the data[S5–S7]. This is exactly the power of persistent homology for machine learning: it quantifies all of the finest up to the coarsest of topological information in data. A prominently persisting void in the tumor surface may quantify the tumor eccentricity, whereas shorter persisting cycles or voids may quantify handles or lobes on, or thus, textural properties of the tumor surface. In the CT scan, the occurrence of many isolated connected components or voids may indicate a more 'spongy' textured tumor. Especially on images with contrast in which these vessels appear brighter, loops may either surround blood vessels or mark circular structures in the vessel trajectories themselves, as shown in Figure S2b. Our topological features then summarize both the occurrence, sizes, quantities, and the distribution of all these types of structural properties. Note that providing a full list of how topological holes may manifest in tumors is beyond our expertise as well as the scope of this paper, and should be ideally analyzed in future work.

As multisets, persistence diagrams cannot be straightforwardly incorporated into many machine learning models. Various methods have been developed to overcome this issue, as we summarize below.

- Vectorized features of a fixed size can be computed from the persistence diagrams. These can be summary statistics, such as the number of points, or various moments (raw, central, standardized) obtained from their lifetimes. This is the approach we used in this paper. Other examples include Betti curves[S8], or discretizations of the persistence diagram density such as persistent images[S9].
- Various kernel methods have been developed for learning from persistence diagrams, such as the persistence Fisher kernel[S10] and the persistence weighted Gaussian kernel[S11].
- Deep learning variants are recently getting more attention[S12–S15]. These are designed to learn a task optimal representation of the persistence diagrams at hand.

For a good overview of more general vectorized and kernelized methods that are designed to learn from persistence diagrams, compatible with the SCIKIT-LEARN library in Python, we recommend[S16].

In this paper, we focus on vectorized features through summary statistics. This allows us to include interpretable feature selection, and lacks the need of choosing hyperparameters, which would be difficult to optimize for our small data sizes. In this way, we are able to construct and evaluate the exact same machine learning pipeline to compare both types of features, i.e., radiomic and topological. Nevertheless, we do not claim this type of feature extraction method to be superior to any of the other methods described above.

Filtrations summarizing topological information in lung lesions. From each scan, we obtained the following five to compute topological information from through persistent homology.

(1) The sublevel filtration obtained from the raw pixel values, restricted to the segmented lesion (see also Figures 2b and 2e).
(2) Same as above, but with negated pixel values.
(3) The sublevel filtration obtained from the raw pixel values, restricted to the boundary box of the segmented lesion. The boundary box is determined by the minimal and maximal (x, y, z) coordinates of the segmentation in the original 3D image scan, and includes topological information of lung tissue surrounding the lesion (see also Figures 2c and 2f).
(4) Same as above, but with negated pixel values.
(5) From the given binary lesion segmentation, we first obtained its surface mesh using the marching cubes algorithm[S17]. Consecutively, we computed the Vietoris-Rips filtration on the vertices of the mesh, thus a point cloud in the Euclidean space $\mathbb{R}^3$ (see also Figure 2d). For computational purposes, the persistence diagrams are approximated through the method described in[S18] using 1000 landmark points (Figure 2g). These landmarks are derived using a furthest-point sampling method, where the next furthest point from all current landmarks is iteratively added to the set of landmarks, which is randomly initialized with one landmark. This procedure provides an even distribution of the selected landmarks. It is however sensitive to outliers, but we do not experience these to affect our particular type of data.

Persistent homology computation. Computing persistence diagrams was performed in Python using DIONYSUS (`https://pypi.org/project/dionysus/`) for image pixels, and RIPSER[S19] for point clouds.

Topological features for lung tumor histology prediction, 20 summary statistics of the persistence diagrams were collected into a topological feature vector. First, each birth-death pair (b, d) was transformed

to a *lifespan* $d - b$ and a *midlife* $\frac{b+d}{2}$ in $\mathbb{R}_{>0} \cup \{\infty\}$. These can be interpreted as the prominence, respectively, the location, of a point in the diagram. From each diagram we computed the following statistics.

(1) The minimal birth-time.
(2) The number of infinite lifespans.
(3) The number of finite lifespans.
(4-19) The mean, standard deviation, skewness, kurtosis, first quartile, median, third quartile, and interquartile range of the finite lifespans and finite midlifes.
(20) The entropy of the finite lifespans[S20].

Per scan, this thus resulted in a topological feature vector of size $5 \times 3 \times 20 = 300$. However, the number of infinite lifetimes is always the same for the six diagrams obtained from the images with boundary box pixels, as well as for the three diagrams obtained from the point clouds, which all have one 0-dimensional hole and no higher-dimensional holes that persist indefinitely. Similarly, the lowest birth-time in a diagram of the 0-dimensional holes in a point cloud is always 0. These features were thus omitted, resulting in a final vector of $300 - 6 - 3 - 1 = 290$ topological features per scan.

We observed that in a few cases there were diagrams without any point (b, d) for which $d < \infty$. The summary statistics from the finite lifespans and midlifes are then not straightforwardly defined. In case of the lifespans, we reasoned that any hole that would have been born, died immediately. The statistics for the finite lifespans were then always defined to be 0, analogous to how they would be defined for a random variable that always evaluates to 0. However, an analogous interpretation for the finite midlifes is more difficult. We therefore treated their summary statistics as missing values.

Note that there may be overlapping information in the persistence diagrams obtained from the filtration of the original and negated image, albeit in different dimensions. For example, voids in one filtration may correspond to connected components in the other (Figure S2b). Therefore, an effective feature selection method as we used in our pipeline, is of crucial importance.

Hyperparameters and settings. For all considered models, we used their standard settings from the Python libraries SCIKIT-LEARN and XGBOOST, apart from changing the output to be probabilistic if needed, i.e., to obtain ROC AUC scores.

References

[S1] A. Hatcher, Algebraic topology, Cambridge University Press, 2002.
[S2] T. Kaczynski, K. M. Mischaikow, M. Mrozek, Computational homology, Vol. 3, Springer, 2004.
[S3] D. Ziou, M. Allili, Generating cubical complexes from image data and computation of the euler number, Pattern Recognition 35 (12) (2002) 2833–2839.
[S4] C. Dang, Triangulations and simplicial methods, Vol. 421, Springer Science & Business Media, 2012.
[S5] O. Dunaeva, H. Edelsbrunner, A. Lukyanov, M. Machin, D. Malkova, R. Kuvaev, S. Kashin, The classification of endoscopy images with persistent homology, Pattern Recognition Letters 83 (2016) 13–22.
[S6] N. Kadoya, S. Tanaka, T. Kajikawa, S. Tanabe, K. Abe, Y. Nakajima, T. Yamamoto, N. Takahashi, K. Takeda, S. Dobashi, et al., Homology-based radiomic features for prediction of the prognosis of lung cancer based on ct-based radiomics, Medical Physics 47 (5) (2020) 2197–2205.
[S7] M. Wang, Z. Cang, G.-W. Wei, A topology-based network tree for the prediction of protein–protein binding affinity changes following mutation, Nature Machine Intelligence 2 (2) (2020) 116–123.
[S8] Y. Umeda, Time series classification via topological data analysis, Information and Media Technologies 12 (2017) 228–239.
[S9] H. Adams, T. Emerson, M. Kirby, R. Neville, C. Peterson, P. Shipman, S. Chepushtanova, E. Hanson, F. Motta, L. Ziegelmeier, Persistence images: A stable vector representation of persistent homology, Journal of Machine Learning Research 18 (2017).
[S10] T. Le, M. Yamada, Persistence fisher kernel: A riemannian manifold kernel for persistence diagrams, arXiv preprint arXiv:1802.03569 (2018).
[S11] G. Kusano, Y. Hiraoka, K. Fukumizu, Persistence weighted gaussian kernel for topological data analysis, in: International Conference on Machine Learning, PMLR, 2016, pp. 2004–2013.
[S12] C. Hofer, R. Kwitt, M. Niethammer, A. Uhl, Deep learning with topological signatures, in: I. Guyon, U. V. Luxburg, S. Bengio, H. Wallach, R. Fergus, S. Vishwanathan, R. Garnett (Eds.), Advances in Neural Information Processing Systems, Vol. 30, Curran Associates, Inc., 2017.
[S13] M. Zaheer, S. Kottur, S. Ravanbakhsh, B. Poczos, R. R. Salakhutdinov, A. J. Smola, Deep sets, in: I. Guyon, U. V. Luxburg, S. Bengio, H. Wallach, R. Fergus, S. Vishwanathan, R. Garnett (Eds.), Advances in Neural Information Processing Systems, Vol. 30, Curran Associates, Inc., 2017.
[S14] M. Carrière, F. Chazal, Y. Ike, T. Lacombe, M. Royer, Y. Umeda, Perslay: a neural network layer for persistence diagrams and new graph topological signatures, in: International Conference on Artificial Intelligence and Statistics, PMLR, 2020, pp. 2786–2796.

[S15] M. Carriere, F. Chazal, M. Glisse, Y. Ike, H. Kannan, Y. Umeda, Optimizing persistent homology based functions, in: International Conference on Machine Learning, PMLR, 2021, pp. 1294–1303.

[S16] The GUDHI Project, GUDHI User and Reference Manual, 3.4.1 Edition, GUDHI Editorial Board, 2021. URL `https://gudhi.inria.fr/doc/3.4.1/`

[S17] W. E. Lorensen, H. E. Cline, Marching cubes: A high resolution 3d surface construction algorithm, ACM siggraph computer graphics 21 (4) (1987) 163–169.

[S18] N. Cavanna, M. Jahanseir, D. Sheehy, A geometric perspective on sparse filtrations, arXiv preprint arXiv:1506.03797 (2015).

[S19] C. Tralie, N. Saul, R. Bar-On, Ripser.py: A lean persistent homology library for python, The Journal of Open Source Software 3 (29) (2018) 925.

[S20] E. Merelli, M. Rucco, P. Sloot, L. Tesei, Topological characterization of complex systems: Using persistent entropy, Entropy 17 (10) (2015) 6872–6892.

Supplemental items

Performances for benign vs. malignant (classification, SF/PA, with contrast)

model	rad	top	concat	vote	stack
LR	86.7 ± 8.7	87.5 ± 10.9	87.2 ± 10.7	$\mathbf{88.9 \pm 9.2}$	88.9 ± 9.2
RF	85.7 ± 12.0	87.9 ± 11.0	87.5 ± 11.6	$\mathbf{88.8 \pm 11.4}$	86.6 ± 11.6
KNN	83.9 ± 11.5	87.2 ± 10.6	87.8 ± 10.0	$\mathbf{87.9 \pm 10.6}$	83.7 ± 10.5
SV	84.4 ± 9.9	85.0 ± 11.6	84.4 ± 11.7	$\mathbf{88.0 \pm 9.5}$	87.0 ± 9.6
BAY	84.9 ± 10.3	85.8 ± 11.9	86.7 ± 12.1	$\mathbf{87.6 \pm 9.5}$	87.2 ± 9.8
XGB	81.9 ± 12.8	$\mathbf{87.6 \pm 12.3}$	86.6 ± 13.4	86.6 ± 11.6	81.5 ± 14.6
mean	84.6 ± 11.0	86.8 ± 11.5	86.7 ± 11.7	$\mathbf{87.9 \pm 10.4}$	85.8 ± 11.3

Table S1. ROC AUC performances in % with standard deviations for *benign vs. malignant* classification of lung tumor CT scan images *with added contrast*, using radiomic features (*rad*) and topological features (*top*), as well as for three models combining both: through concatenation (*concat*), soft voting (*vote*), and stacking. Each scores is averaged over 50 models, obtained through 10-repeated stratified samplings in 5 folds. Best scores are marked in bold.

Performances for benign vs. malignant (classification, SF/PA, without contrast)

model	rad	top	concat	vote	stack
LR	75.6 ± 10.7	77.7 ± 10.0	79.0 ± 9.1	$\mathbf{80.2 \pm 9.6}$	79.7 ± 9.5
RF	72.7 ± 8.8	76.3 ± 8.9	75.9 ± 9.8	$\mathbf{77.9 \pm 9.3}$	69.5 ± 9.7
KNN	72.3 ± 10.3	75.4 ± 9.8	75.5 ± 9.6	$\mathbf{77.8 \pm 9.7}$	72.8 ± 10.1
SV	74.5 ± 10.8	76.2 ± 10.1	78.1 ± 9.4	$\mathbf{78.9 \pm 9.9}$	78.5 ± 10.1
BAY	$\mathbf{78.7 \pm 9.7}$	74.2 ± 10.6	74.7 ± 10.4	77.9 ± 9.1	76.5 ± 9.8
XGB	70.0 ± 10.2	74.7 ± 11.0	75.5 ± 11.0	$\mathbf{76.5 \pm 9.8}$	65.6 ± 12.7
mean	74.0 ± 10.5	75.7 ± 10.1	76.5 ± 10.0	$\mathbf{78.2 \pm 9.6}$	73.8 ± 11.5

Table S2. ROC AUC performances in % with standard deviations for *benign vs. malignant* classification of lung tumor CT scan images *without added contrast*, using radiomic features (*rad*) and topological features (*top*), as well as for three models combining both: through concatenation (*concat*), soft voting (*vote*), and stacking. Each scores is averaged over 50 models, obtained through 10-repeated stratified samplings in 5 folds. Best scores are marked in bold.

Feature correlation for benign vs. malignant (classification, SF/PA, with contrast)

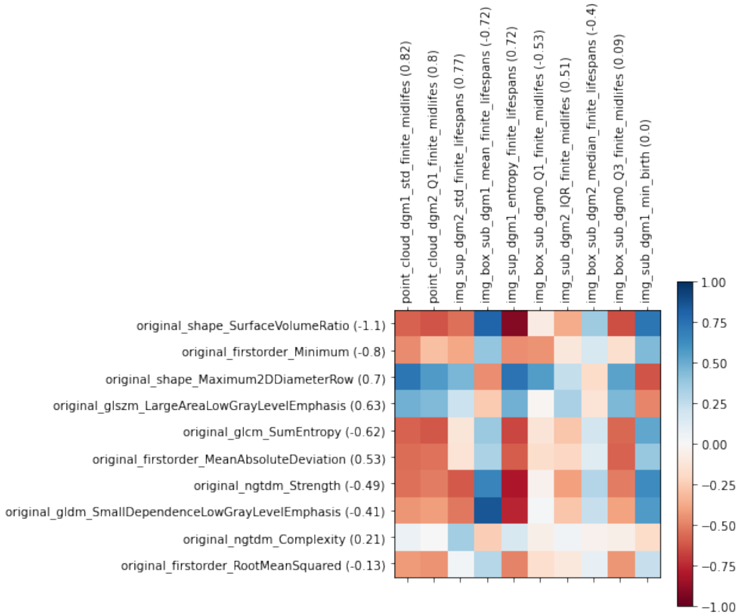

Figure S3. Correlation between the radiomic and topological features selected by the LR models for benign vs. malignant classification with contrast (SF/PA).

Feature correlation for benign vs. malignant (classification, SF/PA, without contrast)

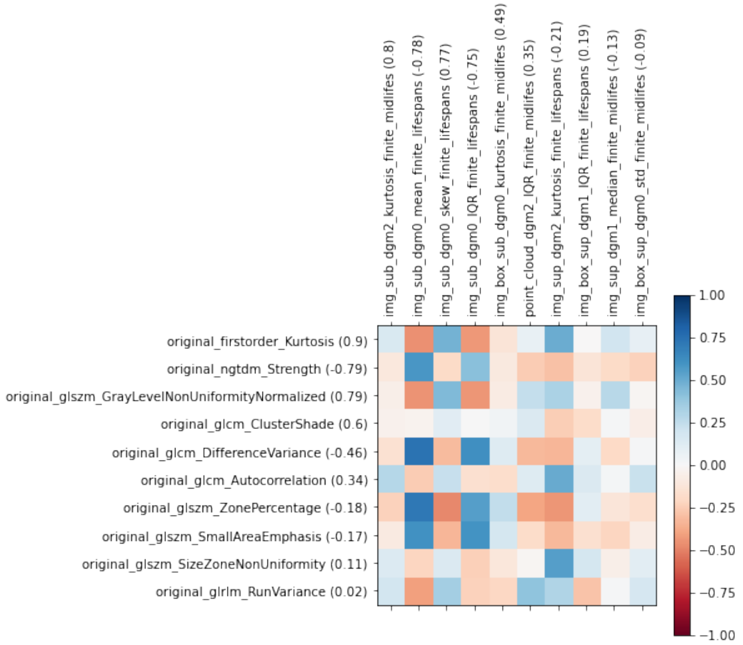

Figure S4. Correlation between the radiomic and topological features selected by the LR models for benign vs. malignant classification without contrast (SF/PA).

Performances for small-cell vs. non-small cell (classification, SF/PA, with contrast)

model	rad	top	concat	vote	stack
LR	**79.8 ± 18.1**	62.6 ± 19.7	62.1 ± 20.9	75.7 ± 19.6	74.4 ± 21.1
RF	**78.4 ± 18.8**	63.2 ± 19.6	73.8 ± 17.7	76.0 ± 19.4	77.2 ± 20.0
KNN	76.8 ± 17.3	64.2 ± 19.5	66.8 ± 19.3	**77.2 ± 18.9**	74.8 ± 19.1
SV	**79.8 ± 18.1**	62.6 ± 18.4	60.8 ± 20.8	75.1 ± 19.5	69.1 ± 25.7
BAY	**73.7 ± 20.6**	62.5 ± 18.4	61.5 ± 18.4	72.8 ± 19.10	64.9 ± 26.5
XGB	**76.4 ± 17.2**	61.4 ± 22.0	71.8 ± 20.6	73.3 ± 17.1	69.7 ± 21.3
mean	**77.5 ± 18.5**	62.7 ± 19.7	66.1 ± 20.3	75.0 ± 19.0	71.7 ± 22.8

Table S3. ROC AUC performances in % with standard deviations for *small cell vs. non-small cell* classification of lung tumor CT scan images *with added contrast*, using radiomic features (*rad*) and topological features (*top*), as well as for three models combining both: through concatenation (*concat*), soft voting (*vote*), and stacking. Each scores is averaged over 50 models, obtained through 10-repeated stratified samplings in 5 folds. Best scores are marked in bold.

Performances for small-cell vs. non-small cell (classification, SF/PA, without contrast)

model	rad	top	concat	vote	stack
LR	80.4 ± 21.5	79.8 ± 19.2	81.6 ± 20.0	**82.6 ± 19.7**	81.6 ± 20.2
RF	83.8 ± 18.9	81.7 ± 19.3	84.4 ± 19.4	**86.8 ± 17.3**	83.8 ± 17.6
KNN	79.1 ± 18.4	78.3 ± 19.0	81.3 ± 18.6	**83.6 ± 19.3**	71.8 ± 24.0
SV	77.9 ± 21.4	77.3 ± 20.2	80.8 ± 21.7	**81.4 ± 20.8**	66.1 ± 29.9
BAY	82.3 ± 19.6	75.9 ± 24.2	77.2 ± 21.2	82.1 ± 19.2	**82.9 ± 19.8**
XGB	80.2 ± 20.1	78.8 ± 22.3	80.3 ± 20.2	**84.1 ± 17.5**	69.5 ± 27.8
mean	80.6 ± 20.1	78.6 ± 20.9	80.9 ± 20.3	**83.4 ± 19.1**	75.9 ± 24.7

Table S4. ROC AUC performances in % with standard deviations for *small cell vs. non-small cell* classification of lung tumor CT scan images *without added contrast*, using radiomic features (*rad*) and topological features (*top*), as well as for three models combining both: through concatenation (*concat*), soft voting (*vote*), and stacking. Each scores is averaged over 50 models, obtained through 10-repeated stratified samplings in 5 folds. Best scores are marked in bold.

Feature correlation for small-cell vs. non-small cell (classification, SF/PA, with contrast)

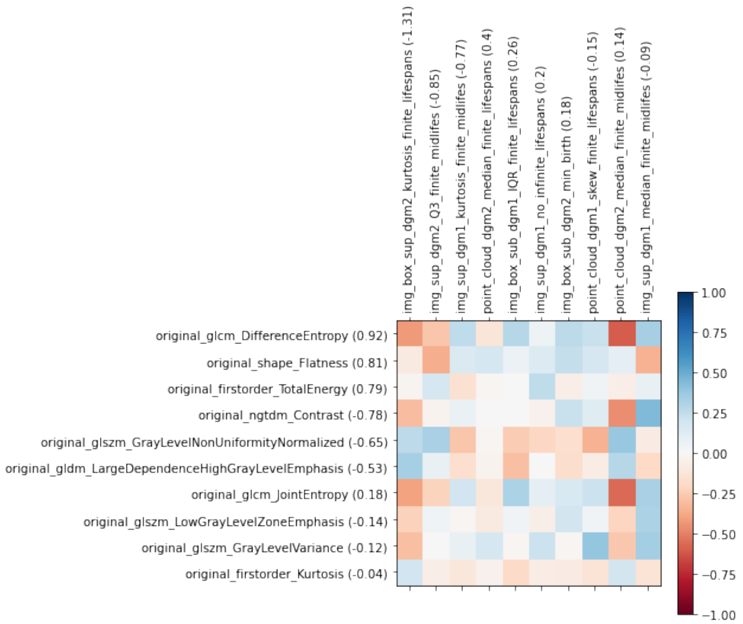

Figure S5. Correlation between the radiomic and topological features selected by the LR models for small-cell vs. non-small cell classification with contrast (SF/PA).

Feature correlation for small-cell vs. non-small cell (classification, SF/PA, without contrast)

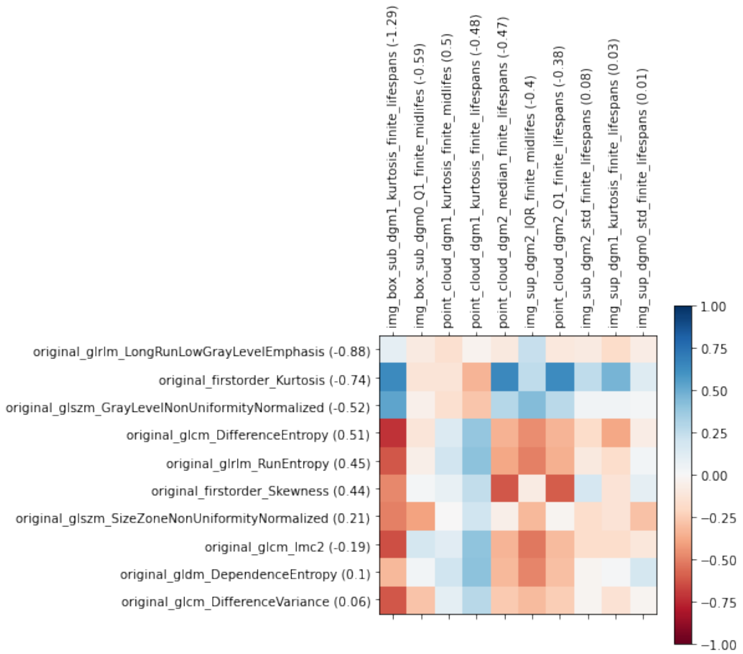

Figure S6. Correlation between the radiomic and topological features selected by the LR models for small-cell vs. non-small cell classification without contrast (SF/PA).

Performances for adeno vs. squamous (classification, SF/PA, with contrast)

model	rad	top	concat	vote	stack
LR	80.2 ± 28.0	$\mathbf{97.3 \pm 10.7}$	$\mathbf{97.3 \pm 10.7}$	96.3 ± 16.1	-
RF	66.8 ± 37.6	$\mathbf{98.3 \pm 8.3}$	$\mathbf{98.3 \pm 8.3}$	91.0 ± 21.9	-
KNN	54.5 ± 25.9	$\mathbf{89.7 \pm 21.6}$	87.2 ± 21.2	86.0 ± 25.1	-
SV	68.5 ± 33.2	94.4 ± 18.4	$\mathbf{97.0 \pm 11.9}$	96.7 ± 15.3	-
BAY	65.5 ± 32.9	70.8 ± 27.1	65.5 ± 29.5	$\mathbf{73.0 \pm 34.2}$	-
XGB	67.2 ± 37.0	96.8 ± 11.2	95.2 ± 14.3	86.8 ± 23.5	-
mean	67.2 ± 33.6	$\mathbf{91.2 \pm 20.0}$	90.1 ± 21.1	88.3 ± 24.8	-

Table S5. ROC AUC performances in % with standard deviations for *adeno vs. squamous* classification of lung tumor CT scan images *with added contrast*, using radiomic features (*rad*) and topological features (*top*), as well as for three models combining both: through concatenation (*concat*), soft voting (*vote*), and stacking. Each scores is averaged over 50 models, obtained through 10-repeated stratified samplings in 5 folds. Best scores are marked in bold. Note that there were insufficient examples of squamous tumors to train a stacking classifier.

Performances for adeno vs. squamous (classification, SF/PA, without contrast)

model	rad	top	concat	vote	stack
LR	62.9 ± 22.4	71.9 ± 16.9	70.7 ± 17.8	$\mathbf{72.1 \pm 18.6}$	69.9 ± 17.9
RF	63.8 ± 25.5	67.8 ± 13.9	66.8 ± 17.6	$\mathbf{70.6 \pm 15.8}$	60.6 ± 23.1
KNN	64.3 ± 19.4	71.0 ± 15.4	69.8 ± 16.3	$\mathbf{74.2 \pm 16.4}$	67.9 ± 18.0
SV	65.9 ± 22.0	71.4 ± 17.3	$\mathbf{71.7 \pm 16.9}$	71.6 ± 17.7	68.3 ± 19.6
BAY	70.0 ± 22.3	69.7 ± 16.9	69.5 ± 16.3	$\mathbf{75.0 \pm 21.7}$	72.2 ± 21.6
XGB	58.8 ± 24.5	$\mathbf{68.4 \pm 14.2}$	64.6 ± 16.6	63.5 ± 20.0	51.8 ± 20.9
mean	64.3 ± 23.0	70.0 ± 15.9	68.8 ± 17.1	$\mathbf{71.2 \pm 18.9}$	65.1 ± 21.4

Table S6. ROC AUC performances in % with standard deviations for *adeno vs. squamous* classification of lung tumor CT scan images *without added contrast*, using radiomic features (*rad*) and topological features (*top*), as well as for three models combining both: through concatenation (*concat*), soft voting (*vote*), and stacking. Each scores is averaged over 50 models, obtained through 10-repeated stratified samplings in 5 folds. Best scores are marked in bold.

Feature correlation for adeno vs. squamous (classification, SF/PA, with contrast)

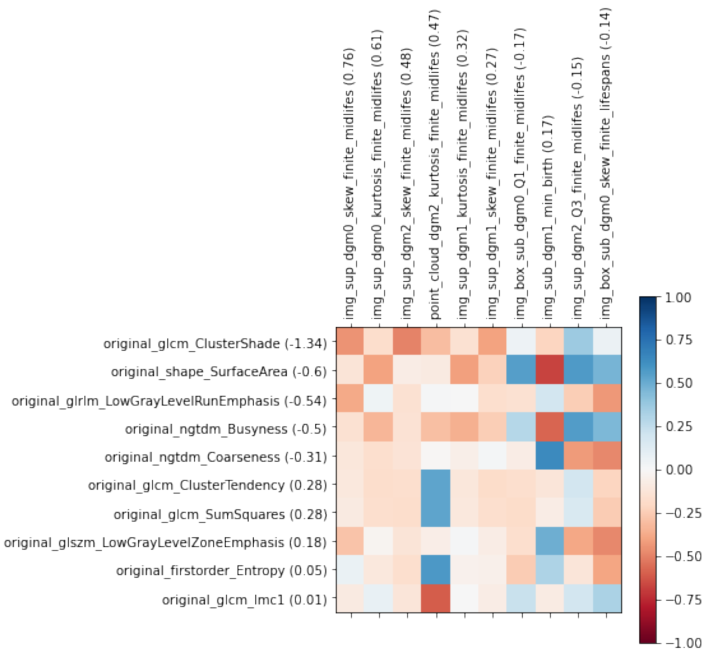

FIGURE S7. Correlation between the radiomic and topological features selected by the LR models for adeno vs. squamous classification with contrast (SF/PA).

Feature correlation for adeno vs. squamous (classification, SF/PA, without contrast)

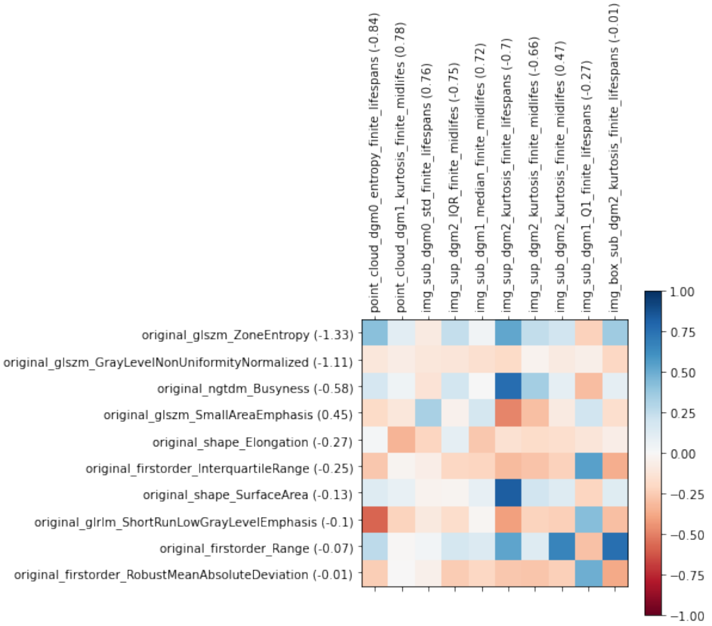

FIGURE S8. Correlation between the radiomic and topological features selected by the LR models for adeno vs. squamous classification without contrast (SF/PA).

Performances for malignancy prediction (regression, LIDC, with contrast)

model	sem	rad	top	concat	vote	stack
LR	61.3 ± 6.4	57.5 ± 6.1	53.3 ± 5.6	53.3 ± 5.5	58.5 ± 5.5	$\mathbf{58.6 \pm 5.7}$
RF	65.3 ± 6.4	57.3 ± 7.0	54.1 ± 7.0	55.5 ± 6.3	$\mathbf{61.3 \pm 6.1}$	52.6 ± 6.8
KNN	61.6 ± 6.9	57.5 ± 6.4	48.7 ± 10.1	51.8 ± 8.7	$\mathbf{59.4 \pm 6.5}$	51.9 ± 7.3
SV	59.9 ± 7.5	56.4 ± 7.1	51.1 ± 6.0	52.4 ± 5.8	57.5 ± 6.1	$\mathbf{57.6 \pm 6.5}$
BAY	61.4 ± 6.3	57.6 ± 5.9	53.5 ± 5.6	53.6 ± 5.4	58.5 ± 5.5	$\mathbf{58.6 \pm 5.8}$
XGB	57.0 ± 7.3	51.3 ± 9.2	51.4 ± 8.0	53.5 ± 7.1	$\mathbf{59.0 \pm 7.1}$	41.9 ± 8.4
mean	61.1 ± 7.2	56.3 ± 7.4	52.0 ± 7.5	53.4 ± 6.7	$\mathbf{59.0 \pm 6.3}$	53.5 ± 9.0

TABLE S7. r^2 performances in % with standard deviations for continuous *malignancy* outcome prediction of lung tumor nodules from CT scan images *with added contrast*, using semantic features (*sem*), radiomic features (*rad*) and topological features (*top*), as well as for three models combining both: through concatenation (*concat*), soft voting (*vote*), and stacking. Each scores is averaged over 50 models, obtained through 10-repeated samplings in 5 folds. Non-semantic best scores are marked in bold.

Performances for malignancy prediction (regression, LIDC, without contrast)

model	sem	rad	top	concat	vote	stack
LR	54.8 ± 4.9	43.3 ± 5.5	35.6 ± 7.1	36.4 ± 6.9	44.2 ± 5.2	$\mathbf{45.1 \pm 5.3}$
RF	56.9 ± 5.1	45.3 ± 6.0	41.0 ± 8.0	43.6 ± 6.4	$\mathbf{49.0 \pm 5.6}$	36.4 ± 7.9
KNN	54.2 ± 6.1	39.6 ± 7.3	31.8 ± 9.9	35.4 ± 9.4	$\mathbf{45.2 \pm 6.2}$	34.5 ± 8.2
SV	54.1 ± 5.1	42.1 ± 5.8	34.8 ± 7.2	35.5 ± 7.3	43.8 ± 5.4	$\mathbf{44.3 \pm 5.6}$
BAY	54.8 ± 4.9	43.5 ± 5.4	35.7 ± 7.0	36.6 ± 6.9	44.1 ± 5.2	$\mathbf{45.1 \pm 5.3}$
XGB	50.6 ± 5.3	43.0 ± 6.4	39.6 ± 8.1	41.7 ± 6.6	$\mathbf{48.3 \pm 5.7}$	27.4 ± 11.4
mean	54.2 ± 5.6	42.8 ± 6.3	36.4 ± 8.5	38.2 ± 8.0	$\mathbf{45.8 \pm 6.0}$	38.9 ± 10.1

TABLE S8. r^2 performances in % with standard deviations for continuous *malignancy* outcome prediction of lung tumor nodules from CT scan images *without added contrast*, using semantic features (*sem*), radiomic features (*rad*) and topological features (*top*), as well as for three models combining both: through concatenation (*concat*), soft voting (*vote*), and stacking. Each scores is averaged over 50 models, obtained through 10-repeated samplings in 5 folds. Non-semantic best scores are marked in bold.

Feature correlation for malignancy prediction (regression, LIDC, with contrast)

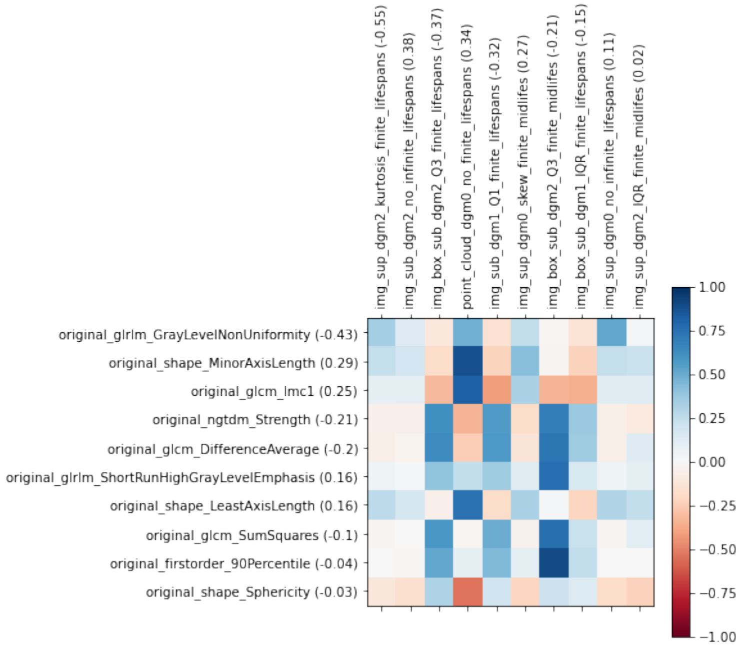

Figure S9. Correlation between the radiomic and topological features selected by the LR models for malignancy prediction with contrast (regression, LIDC).

Feature correlation for malignancy prediction (regression, LIDC, without contrast)

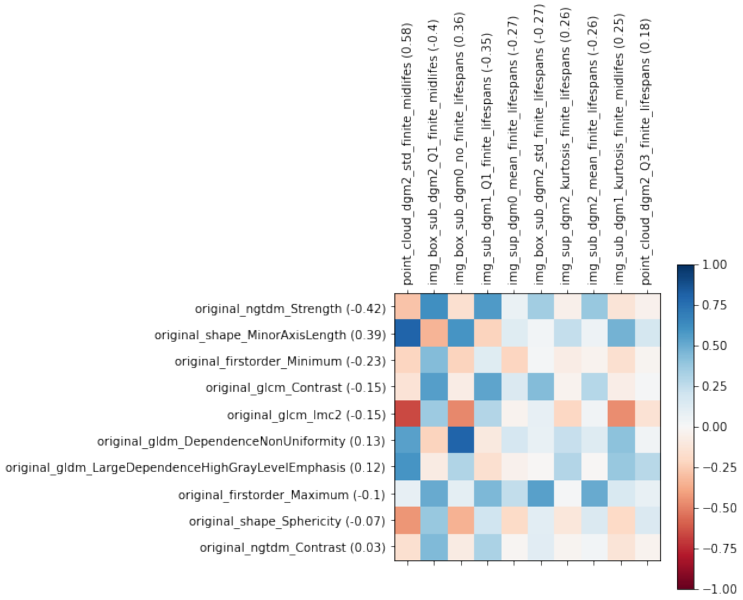

Figure S10. Correlation between the radiomic and topological features selected by the LR models for malignancy prediction without contrast (regression, LIDC).

Performances for benign vs. malignant (classification, LIDC, with contrast)

model	sem	rad	top	concat	vote	stack
LR	66.3 ± 20.2	53.6 ± 19.3	$\mathbf{60.3 \pm 18.9}$	56.4 ± 17.5	57.5 ± 20.2	54.2 ± 20.7
RF	68.2 ± 19.3	57.0 ± 18.4	$\mathbf{61.0 \pm 20.2}$	59.3 ± 19.4	60.6 ± 18.0	50.0 ± 22.1
KNN	66.8 ± 20.1	66.3 ± 17.8	61.7 ± 19.0	62.4 ± 18.6	64.5 ± 18.7	$\mathbf{67.7 \pm 16.4}$
SV	67.7 ± 20.8	56.7 ± 20.6	$\mathbf{59.3 \pm 19.8}$	57.0 ± 15.0	53.9 ± 20.7	52.8 ± 19.7
BAY	64.3 ± 19.5	57.4 ± 20.3	$\mathbf{61.9 \pm 19.0}$	59.7 ± 18.8	61.3 ± 19.4	55.3 ± 22.3
XGB	68.0 ± 16.3	57.9 ± 17.6	$\mathbf{65.7 \pm 17.4}$	61.1 ± 17.8	63.0 ± 17.5	59.5 ± 21.5
mean	66.9 ± 19.5	58.2 ± 19.4	$\mathbf{61.6 \pm 19.2}$	59.3 ± 18.0	60.1 ± 19.5	56.6 ± 21.3

Table S9. ROC AUC performances in % with standard deviations for *benign vs. malignant* classification of lung tumor nodules from CT scan images *with added contrast*, using semantic features (*sem*), radiomic features (*rad*) and topological features (*top*), as well as for three models combining both: through concatenation (*concat*), soft voting (*vote*), and stacking. Each scores is averaged over 50 models, obtained through 10-repeated samplings in 5 folds. Non-semantic best scores are marked in bold.

Performances for benign vs. malignant (classification, LIDC, without contrast)

model	sem	rad	top	concat	vote	stack
LR	16.0 ± 35.3	58.3 ± 46.2	62.0 ± 43.1	$\mathbf{70.3 \pm 42.3}$	53.0 ± 47.7	43.7 ± 43.2
RF	9.3 ± 21.4	57.3 ± 42.6	59.7 ± 41.4	$\mathbf{65.3 \pm 42.7}$	60.0 ± 42.6	24.7 ± 37.0
KNN	29.3 ± 34.6	45.8 ± 33.9	58.0 ± 36.9	$\mathbf{59.5 \pm 37.7}$	52.7 ± 44.7	45.0 ± 32.5
SV	12.0 ± 30.9	$\mathbf{64.3 \pm 45.8}$	56.0 ± 43.2	63.7 ± 43.8	49.0 ± 45.3	51.3 ± 41.3
BAY	14.8 ± 26.2	57.7 ± 40.3	66.5 ± 32.3	64.5 ± 30.5	$\mathbf{76.2 \pm 35.1}$	46.2 ± 43.1
XGB	12.3 ± 21.6	40.8 ± 33.2	76.5 ± 34.8	74.0 ± 38.4	$\mathbf{78.0 \pm 34.9}$	49.0 ± 36.7
mean	15.6 ± 29.6	54.1 ± 41.5	63.1 ± 39.4	$\mathbf{66.2 \pm 39.8}$	61.5 ± 43.5	43.3 ± 40.1

Table S10. ROC AUC performances in % with standard deviations for *benign vs. malignant* classification of lung tumor nodules from CT scan images *without added contrast*, using semantic features (*sem*), radiomic features (*rad*) and topological features (*top*), as well as for three models combining both: through concatenation (*concat*), soft voting (*vote*), and stacking. Each scores is averaged over 50 models, obtained through 10-repeated samplings in 5 folds. Non-semantic best scores are marked in bold.

Feature correlation for benign vs. malignant (classification, LIDC, with contrast)

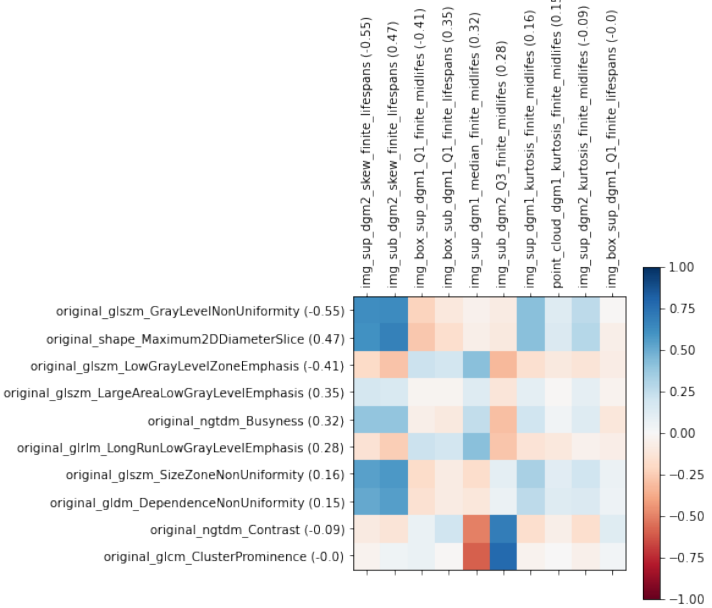

Figure S11. Correlation between the radiomic and topological features selected by the LR models for benign vs. malignant classification with contrast (LIDC).

Feature correlation for benign vs. malignant (classification, LIDC, without contrast)

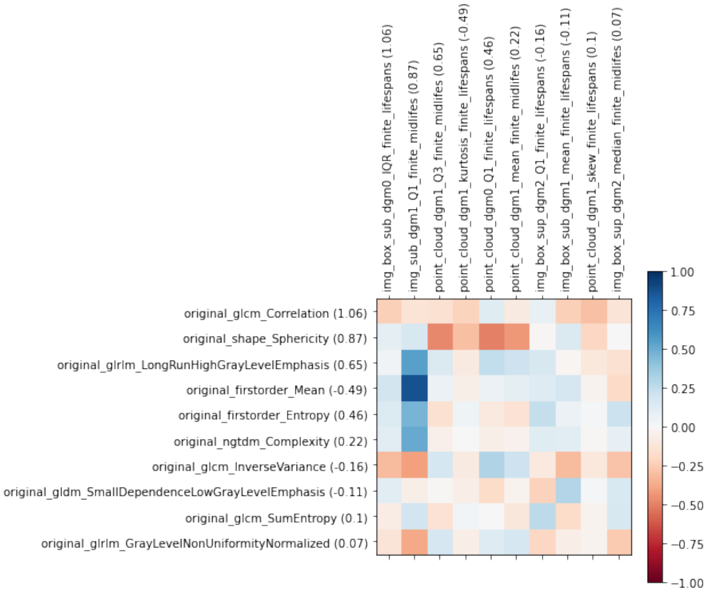

Figure S12. Correlation between the radiomic and topological features selected by the LR models for benign vs. malignant classification without contrast (LIDC).
